# Trapping X‐ray Radiation Damage from Homolytic Se−C Bond Cleavage in BnSeSeBn Crystals (Bn=benzyl, CH_2_C_6_H_5_)

**DOI:** 10.1002/anie.202203665

**Published:** 2022-05-03

**Authors:** Christian J. Schürmann, Thorsten L. Teuteberg, A. Claudia Stückl, Paul Niklas Ruth, Fabian Hecker, Regine Herbst‐Irmer, Ricardo A. Mata, Dietmar Stalke

**Affiliations:** ^1^ Georg-August Universität Göttingen Institut für Anorganische Chemie Tammannstraße 4 37077 Göttingen Germany; ^2^ Georg-August Universität Göttingen Institut für Physikalische Chemie Tammannstraße 2 37077 Göttingen Germany; ^3^ Max-Planck-Institut für Biophysikalische Chemie Am Fassberg 11 37077 Göttingen Germany

**Keywords:** Benzyl Radicals, Charge Density Determination, Radicals, Selenium, X-ray Damage

## Abstract

Irradiation of dibenzyl diselenide BnSeSeBn with X‐ray or UV‐light cleaves the Se−C and the Se−Se bonds, inducing stable and metastable radical states. They are inevitably important to all natural and life sciences. Structural changes due to X‐ray‐induced Se−C bond‐cleavage could be pin‐pointed in various high‐resolution X‐ray diffraction experiments for the first time. Extended DFT methods were applied to characterize the solid‐state structure and support the refinement of the observed residuals as contributions from the BnSeSe⋅ radical species. The X‐ray or UV‐irradiated crystalline samples of BnSeSeBn were characterized by solid‐state EPR. This paper provides insight that in the course of X‐ray structure analysis of selenium compounds not only organo‐selenide radicals like RSe⋅ may occur, but also organo diselenide BnSeSe⋅ radicals and organic radicals R⋅ are generated, particularly important to know in structural biology.

## Graphical abstract

Irradiation of dibenzyl diselenide BnSeSeBn with X‐ray or UV‐light homolytically cleaves the Se−C and Se−Se bonds inducing stable and metastable radical states. Structural changes due to X‐ray‐induced Se−C bond‐cleavage could be pin‐pointed in various high‐resolution X‐ray diffraction experiments.

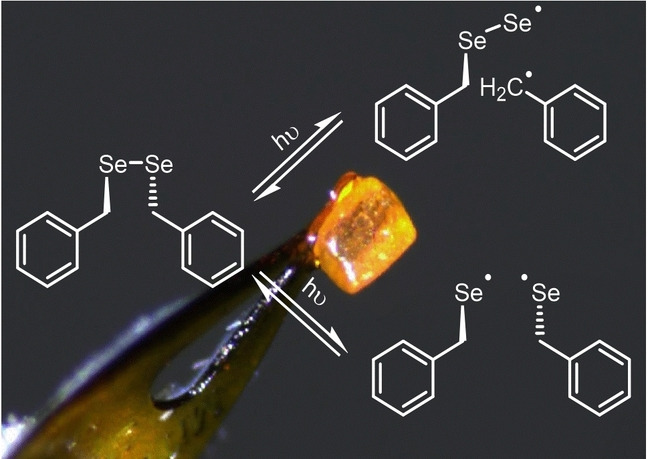

The interaction of organo selenium compounds with radiation is interdisciplinarily appreciated in natural sciences: In chemistry, they exhibit a vivid radical reactivity and frequently serve as radical precursors, easily accessible by photochemical or thermal activation of either the Se−Se or Se−C bonds.^[1]^ In physics, selenium shows a K_α_ fluorescence emission line^[2]^ at 12.7 keV with a quantum yield of 0.567. Furthermore, it is known to be a semiconductor, applied in X‐ray imaging.^[3]^ In structural biology selenium is introduced into macromolecular structures for various phasing methods in structure solution:^[4]^ While (Multi‐wavelength Anomalous Dispersion) MAD‐phasing^[5]^ makes use of the relatively strong anomalous signal of selenium, (Radiation‐damage Induced Phasing) RIP^[6]^ uses the defects, introduced by radiation damage, predominantly unspecific at selenium sites, and in UV‐RIP,^[7]^ radiation damage is intentionally introduced at these sites by UV‐irradiation.^[8]^ The underlying mechanism of this specific radiation damage is anticipated to be a relatively unspecific radical pathway.^[9]^ For dibenzyl diselenide BnSeSeBn (**1**), studied in this paper, the chemical reactivity manifests itself in the photolytic formation of radicals in the solid state[^10^, ^11^, ^12^] and in solution.^[13]^ A disproportionation reaction gives ultimately elemental selenium and benzaldehyde (in the presence of O_2_)^[14]^ or dibenzyl selenide (when O_2_ is excluded)^[15]^ when exposed to UV‐ or sunlight. In both cases, the proposed reaction pathway begins with the photoinduced homolytic dissociation of either the Se−C bond, to give BnSeSe⋅/⋅Bn (**2**), and/or the Se−Se bond, to give two equivalents BnSe⋅ (**3**),^[16]^ leading to a metastable bi‐radical state (Scheme 1). Bond cleavage and radical formation upon X‐ray or UV‐radiation is known for various small molecules.^[17]^

**Scheme 1:**
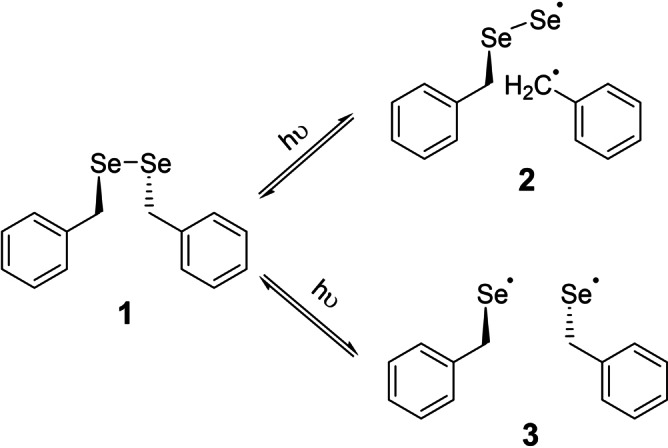
Photolytic dissociation of the diselenide BnSeSeBn (**1**) into BnSeSe⋅/⋅Bn (**2**) and two BnSe⋅ (**3**) radicals by either homolytic Se−C or Se−Se bond cleavage

The solid‐state structure of BnSeSeBn (**1**) is fascinating on its own, showing the Bn‐groups in *gauche*‐formation, featuring intermolecular Se⋅⋅⋅Se distances of only 3.4425(1) Å, and forming intermolecular strands of Se_*n*_ along the crystallographic c‐axis (Figures 1a, S1 and S2), different to the helical arrangement in grey selenium. Particularly these weak chalcogen–chalcogen interactions render the compound interesting for e.g. band gap tuning in the solid molecular alloys of *x*(PhSeSePh)/1−*x*(PhTeTePh).^[18]^ The relatively high percentage of the selenium content of the structure results in high X‐ray fluorescence intensity (Figure S3), thus the collection of suitable high‐resolution X‐ray diffraction data for experimental charge‐density studies proved challenging. Therefore, six datasets were collected at different in‐house diffractometers (Tables 1 and S1 to S7) with various X‐ray wavelengths, intensities and detectors at 100 K.^[19]^ Subsequently, for all datasets in this paper charge density models were refined in the multipole approach^[20]^ using the XD2006^[21]^ (Table S8, S9 and S11 to S13, Figures S4 and S7 to S30) as well as the MoPro suite^[22]^ (Table S10 and Figure S5) in order to analyze the structure according to Bader's Quantum Theory of Atoms in Molecules (QTAIM).^[23]^ In addition, an extended DFT optimization was applied to gain a good estimation of the theoretical charge density distribution. A hybrid (Quantum Mechanics/Molecular Mechanics) QM/MM model^[24]^ was applied to simulate a single QM molecule in an MM environment. The electrostatic part of the embedding is represented with point charges, which are determined self‐consistently from the QM density in order to ensure a consistent density (Table S14–S25; Figures S31–S33). However, all thoroughly refined models showed an unsatisfactory fit to the corresponding datasets as well as differences to the theoretical electron density distribution in varying degrees (Table S1). Yet one persistent salient feature was found throughout all datasets and refinements: Two relatively high residual density peaks in the proximity of selenium at similar, but initially counterintuitive, positions for all datasets were found (Figure 1b). As this feature is found for different crystals, different refinement approaches and regardless of the experimental setup and exposure time, it can be excluded that they are artefacts due to absorption, twinning, model insufficiencies, X‐ray fluorescence or simply bad crystals and data quality. Additionally, they could by non of the multiple attempted models be interpreted satisfactory as disorder of the molecule.

**Figure 1:**
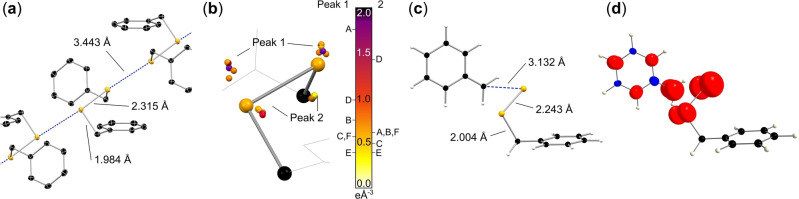
High‐resolution X‐ray structure of BnSeSeBn (**1**) orientated along the Se−Se bonds and Se⋅⋅⋅Se long‐range interactions (a) and the location of the various residual peaks from experiments with various X‐ray sources, scaled by the electron densities from those experiments A to F (b and Table 1). Theoretically confirmed Se−C bond cleavage to give the BnSeSe⋅/⋅Bn (**2**) radicals in the single crystal state (c) as well as the theoretically determined spin density of **2** (d).

**Table 1:** Crystallographic details of all collected datasets (for more details see Supporting Information).

Dataset^[a]^	A	B	C	D	E	F
source power [W]	1200	30	30	1200	70	140
*λ* [Å]	0.71073 (Mo)	0.56086 (Ag)	0.56086 (Ag)	0.56086 (Ag)	0.56086 (Ag)	0.51340 (In)
*μ* [mm^−1^]	5.923	3.129	3.135	3.134	3.133	2.463
Θ_max_ [°]	52.248	38.576	38.663	38.644	38.578	34.803
max. res. [Å]	0.449	0.450	0.449	0.449	0.450	0.450
coll. ref.	12 4340	12 4178	96 429	21 2039	12 5335	12 1728
ind. ref.	7162	7196	7123	7197	7171	7171
R1 (MM) [%]	2.25	1.55	1.51	1.65	1.35	1.82
wR2 (MM) [%]	2.30	1.27	1.49	1.89	1.28	1.42
GOF	4.901	2.027	1.528	5.636	2.123	1.676
Δ*ρ* (F^2^, MM) [eÅ^−3^]	1.603	0.508	0.392	0.992	0.371	0.574
	−0.626	−0.441	−0.248	−0.531	−0.401	−0.310

[a] See also scale at the right in Figure 1b.

In order to quantify the residual peaks and to benchmark the experimental data towards the theoretically expected charge‐density, theoretical multipole populations were determined using DenProp^[25]^ (Figure S34), and applied to all datasets analogous to the invariome approach.^[26]^ Again, the residuals were obvious (Figures S35 to S41). Clearly, their heights correlate with the power of the various sources and hence the intensity of the different primary X‐ray beams (Figures 1b, S42 and Table S25). In view of the photo reactivity of BnSeSeBn the residual density peaks were considered to be caused by a radiation activated form of the molecule. Therefore, an extended DFT optimization of the possible activated states was performed under the additive crystal QM/MM scheme (AC‐QM/MM).^[24]^ The latter allows with minimal added cost to optimise the structure of a defect (in this case a molecule found in an excited state) in the environment of an otherwise unperturbed crystal. Further details are available in the Supporting Information. Based on the optimized ground state geometry, the MM environment was frozen and only the QM molecule was allowed to change its geometry. The QM molecule was optimized in different electronic states in the frozen environment (see Table S14–S24). The triplet state optimization yielded a broken Se−C bond and selenium positions, equivalent to **2**, that closely resemble the locations of the residual density peaks (Figure 1c). Therefore, the observed residual density peaks can be described as the ground state structure **1** superimposed by the activated state structure **2**. The more intense the applied radiation is, the higher the residuals are, hence the more the activated state BnSeSe⋅/⋅Bn (**2**) is populated. Different to literature expectations cleavage of the Se−Se bond and the population of BnSe⋅/⋅SeBn (**3**) is not observed. The occurrence of **2** is of particular concern for X‐ray structure determination in structural biology, particularly using very high flux densities, since the produced organic radical may lead to vivid unforeseen reaction cascades inside the studied biomolecules. When solid BnSeSeBn (**1**) is UV‐irradiated for at least several hours at liquid nitrogen temperature, it yields EPR‐active species.^[10]^ In order to clarify the presence of metastable radicals in X‐ray as well as UV irradiated samples and investigate their nature, we exposed crystalline samples of **1** to the beam of a high‐power rotating Cu X‐ray anode and to light of a 150 W Hg(Xe) arc UV lamp for six hours at 143 and 77 K, respectively. The resulting EPR signals in Figures 2a–c clearly show the emergence of unpaired spins upon X‐ray and UV‐irradiation. All EPR spectra of irradiated samples (Figures 2b–g) feature signals at g= 2.0035 and to a smaller extent also at g=2.0950. The UV‐irradiated sample (Figure 2b) shows a broad symmetric and unresolved pattern at g=2.0039 with a peak‐to‐peak width of 34 G, the typical characteristics of a Bn⋅ radical signal.^[27]^ Additionally, it shows a small feature at the center of the peak, as well as two small broad side peaks with about 10 % of the total signal intensity and an estimated peak‐to‐peak distance of about 86 G. These broad peaks are probably due to coupling of Bn⋅ with Se atoms at a close distance (^77^Se (I=1/2), natural abundance 7,58 %), while the small feature in the center is probably due to a small percentage of Se centered radical signals (vide infra). The X‐ray irradiated sample (Figure 2c–f), on the other hand, features an intense, sharp, asymmetric peak at g=2.0032 showing a slight anisotropy in the centre when measured at very high resolution (Figure 2d), as well as several less resolved satellite peaks. The signal is overlaid by a relatively weak but broad signal of about 30 G peak‐to‐peak distance, close to the Bn⋅ signal characteristics as described. Upon aging, the UV‐irradiated samples’ characteristic Bn⋅ signal depletes completely (Figure S42B), while the EPR signal of the X‐ray irradiated sample only loses the weak, broad, underlying signal and hence becomes much clearer (Figure 2e): two sets of satellite peaks with splittings of 75 and 115 G, respectively, are now observed. The satellites (sets 1 and 2) are symmetrical to slightly different g‐values (g1=2.0032, A1=58 G; g2=2.0040–2.0045, A2=38 G; Figure 2f) and show, in a crude estimation, the following intensity ratio: intensity (set 1) : intensity (set 2)=1 : 2.

**Figure 2:**
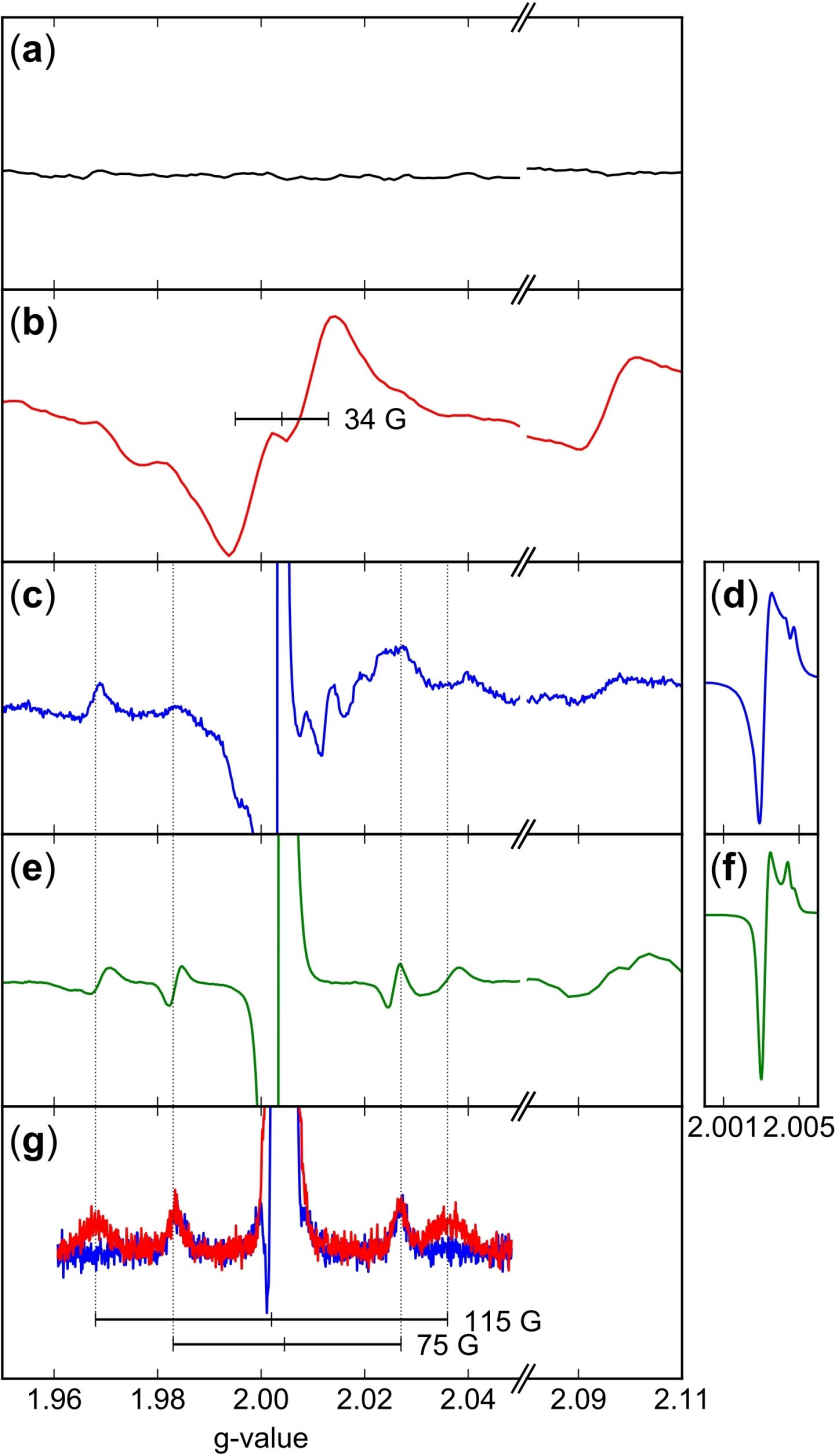
EPR signals of unirradiated (a), UV‐irradiated (b) and X‐ray irradiated (c) crystalline BnSeSeBn (**1**) powder at 143 K. The detailed measurement of the main signal of (d) is shown in (c), EPR signals of X‐ray irradiated sample after 60 days at room temperature (e), the detailed measurement of the main signal of (e) is shown in (f), X band (9 GHz) echo‐detected pulsed EPR spectra of X‐ray irradiated sample after 60 days with different time delays between detection pulses, i.e. 420 ns (red) and 3080 ns (blue). The marked values correspond to the hyperfine splitting (g).

Such signals are consistent with two distinct radical species with different environments, each strongly coupled to nuclear spins I=1/2. The intensity ratio of the peak pattern is consistent with a mixture of Se⋅ (set 1) and Se_2_⋅ (set 2) species.^[28]^ By spin‐echo EPR (Figure 2g), we were able to separate the two components according to their different spin–spin relaxation time *T*_2_ and to confirm the assignment. It is therefore concluded, that the UV‐ as well as the X‐ray irradiated samples feature an overlay of Bn⋅, Se⋅ and Se_2_⋅ radical species, like BnSeSe⋅, in varying proportions: while X‐rays are mainly absorbed at selenium and hence lead to Se‐centered radical species, UV‐light is absorbed at the aromatic moieties and leads to Bn‐centered radicals. While Bn⋅ depletes or reacts with oxygen to benzaldehyde, the selenium‐centered radical species are persistent. This observation was not described before, e.g. in earlier studies of pure Se.^[12]^ Hence, a strong support of the stability of these different Se‐centered radical species by short Se−Se distances and chain‐like ordering of Se atoms, like in crystalline BnSeSeBn, is expected to be the reason. Finally, for both kinds of excitation, signals at g=2.095 are observed, that emerge with a red discoloration in the samples. We therefore assign them to emerging polyselenides, as found by Sampath^[12]^ in contrast to prior studies.^[10]^ A more detailed EPR analysis is given in Figures S42 to S45. Revisiting some charge density investigations of organo selenium compounds proved that this radiation induced Se−C bond cleavage not only occurs in BnSeSeBn (**1**) but is of more general concern. Works of e.g. Espinosa et al.^[29]^ and Ganter, Novaković, Bogdanović et al.^[30]^ prove the charge density investigation of Se‐containing structures to be challenging, as the refinement results show significant, but so‐far unassigned residual density features around selenium. One possible source of these features could be the manifestation of radiation damage and the subsequent formation of radical species.

In conclusion, the multiple high‐resolution multipole refinements of BnSeSeBn (**1**) with various X‐ray intensities and wavelengths unearthed the trapping of a persistent radiation‐induced radical species BnSeSe⋅/⋅Bn (**2**) in the single crystalline state. The related residual peak heights correlate directly with the intensity of the used X‐ray source. Other structural changes apart from the homolytic Se−C bond cleavage are so minute that a phase transition is not observed and the structural periphery is not affected. Hybrid QM/MM calculations confirmed **2** to be a minimum on the energy hyperphase and identified the residual peaks from the diffraction experiments to be the selenium positions of **2**. UV‐ and X‐ray induced radiation damage on the crystalline sample of **1** followed by EPR spectroscopy confirmed at least three radical species to be present, Bn⋅, Se⋅ and Se_2_⋅. This unequivocal proof is particularly important in structural biology, because especially in selenium MAD phasing the present organic radical causes a vivid unforeseen reaction cascade to the protein, i.e. not only resulting in the simple protonation of the related selenium residue, but also might give hydroxygenation, oxidation or C−C bond formation. This is even more likely as current experiments in e.g. structural biology apply very high X‐ray intensities from powerful state‐of‐the‐art synchrotrons. In material science the problem might even be aggravated by long exposure times. In any interpretation and discussion of selenium containing structures it is important to take those radical‐induced processes into account.

Deposition Numbers 2157506, 2157507, 2157508, 2157509, 2157519, 2157511 (for **1**) contain the supplementary crystallographic data for this paper. These data are provided free of charge by the joint Cambridge Crystallographic Data Centre and Fachinformationszentrum Karlsruhe Access Structures service.

## Conflict of interest

The authors declare no conflict of interest.

## Supporting information

As a service to our authors and readers, this journal provides supporting information supplied by the authors. Such materials are peer reviewed and may be re‐organized for online delivery, but are not copy‐edited or typeset. Technical support issues arising from supporting information (other than missing files) should be addressed to the authors.

Supporting Information

Supporting Information

Supporting Information

Supporting Information

Supporting Information

Supporting Information

Supporting Information

Supporting Information

## Data Availability

The data that support the findings of this study are available from the corresponding author upon reasonable request.
