# Supplementary material for: Trapping X‐ray Radiation Damage from Homolytic Se−C Bond Cleavage in BnSeSeBn Crystals (Bn=benzyl, CH2C6H5)
Source: Angew Chem Int Ed Engl. 2022 May 3;61(26):e202203665. doi: 10.1002/anie.202203665 (PMC9320817; doi:10.1002/anie.202203665)
Supplement: Supplementary file 8 — Supporting Information [file ANIE-61-0-s003.pdf]

Angewandte Chemie
Eine Zeitschrift der Gesellschaft Deutscher Chemiker

Supporting Information

Trapping X-ray Radiation Damage from Homolytic Se–C Bond Cleavage in BnSeSeBn Crystals (Bn = benzyl, $CH_2C_6H_5$)

*C. J. Schürmann, T. L. Teuteberg, A. C. Stückl, P. N. Ruth, F. Hecker, R. Herbst-Irmer, R. A. Mata, D. Stalke**

Crystallographic Details

High-resolution diffraction datasets were collected from partially different crystals of $(BnSe)_2$ on different machine setups. All frames were converted into the Bruker *.sfrm* frame format with a Python [1] script. using the Fabio library for reading and matching header entries.[2] and our own implementation for writing .sfrm frames. The frames were integrated with SAINT v8.37A [3] up to a resolution of 0.45 Å. For several datasets – especially those collected with Pilatus3 detector, the SAINT default settings were altered: the integration boxsize (SPOTSIZE, YSPOTSIZE, SPREAD) and the number of points for the peak profile fit (PROFXHALF, PROFYHALF, PROFZHALF). Furthermore, variations in the default values of the strong and weak reflection limits (STRONGTHRESH and LS_IOVS_MAX), the background correlation lengths (BGCORSCALE), and the background determination procedure (PLANEBG) were adopted to some integrations.

Dataset **A** was collected on a Bruker D8 3-circle goniometer, equipped with a 1200W SRA TXS rotating anode (Mo K_α radiation), Montel Mirror optics and a Bruker SMART APEX 2 detector. It was integrated using the PLANEBG option for the background determination.

Dataset **B** was collected on a Bruker D8 3-circle goniometer, equipped with a 30W Incoatec Microfocus Source (IµS) (Ag K_α radiation) and a Bruker SMART APEX 2 detector. It was integrated, using a fixed integration box size (SPOTSIZE = 0.8, YSPOTSIZE = 0.8, SPREAD = 0.5) and a reduced background correlation lengths (BGCORSCALE = -2).

Dataset **C** was collected on a Bruker D8 3-circle goniometer, equipped with a 30 W Incoatec Microfocus Source (IµS) (Ag K_α radiation) and a Dectris Pilatus3 300K CdTe pixel detector. It was integrated with an increased number of profile fitting points (PROFXHALF = 12, PROFYHALF = 12, PROFZHALF = 12) and the refined integration box size resulted in relatively large mean values of SPOTSIZE = 1.280, YSPOTSIZE = 1.471, SPREAD = 0.754). The strong and weak reflection limits were adapted (STRONGTHRESH = 15 and LS_IOVS_MAX = 10). It is assumed that by the extremely low background, the peak broadening by thermally diffuse scattering (TDS) is detected very well and leads to a very broad peak-shape, that is insufficiently described by the standard amount of peak profile points.

Dataset **D** was collected on a Rigaku kappa goniometer, equipped with a 1200 W Rigaku MicroMax 007 (Ag K_α radiation) and a Dectris Pilatus3 300K CdTe pixel detector. It was integrated with a reduced background correlation lengths (BGCORSCALE = -2).

Dataset **E** was collected on a Bruker Venture kappa goniometer, equipped with a 70W Incoatec Microfocus Source (IµS) (Ag K_α radiation) and a Bruker Photon 2 detector. It was integrated using the SAINT defaults.

Dataset **F** was collected on a Bruker Venture kappa goniometer, equipped with a 140 W Excilium Metaljet x-ray source (In K_α radiation), Incoatec mirror optics and a Bruker Photon 2 detector. It was integrated using the SAINT defaults.

The scaling and absorption correction were performed with SADABS. [4] No error model was applied so experimental errors were retained. Data quality statistics were created with XPREP [5]. The structure was solved with SHELXT [6] and the IAM refinement was performed with SHELXL [7], using the GUI ShelXle [8].

The starting models for the charge-density refinements were prepared by the refinement of the heavy atom location and vibrational parameters against high resolution data (< 0.6 Å), while the hydrogen positions were placed at the difference Fourier maxima against low resolution data (> 1.0 Å).

The charge density refinements were performed in the XD [9] (l_{max} = 4) and the MoPro suite [10] (l_{max} = 6). The refinement procedure was developed under consideration of the R_{cross}-value [11], the Probability Density Function (PDF), Kuhs's Rule [12], DRK-plot [13–15] and residual density analysis [16].

Table S1.

Crystallographic details of all collected datasets in the scape group *C*2/c.

Dataset	**A**	**B**	**C**	**D**	**E**	**F**
CCDC for IAM	2157506	2157507	2157508	2157509	2157510	2157511
Detector	Bruker Smart Apex2 Ultra		Dectris Pilatus 3 300K CdTe		Bruker Photon2	
X-ray source	Bruker SRA TXS	IμS	IμS	MicroMax 007	IμS3	MetalJet
Source power [W]	1200	30	30	1200	70	140
λ [Å]	0.71073	0.56086	0.56086	0.56086	0.56086	0.5136
a [Å]	13.7033(10)	13.717(2)	13.7123(18)	13.7199(11)	13.7247(7)	13.7299(6)
b [Å]	8.0012(6)	8.0127(13)	8.0051(11)	8.0119(6)	8.0053(4)	8.0135(4)
c [Å]	11.4769(8)	11.4944(18)	11.4849(15)	11.4766(9)	11.4831(6)	11.4875(5)
β [°]	99.293(2)	99.303(7)	99.260(5)	99.301(2)	99.2776(16)	99.2957(15)
V [$Å^{-3}$]	1241.84(16)	1246.8(3)	1244.3(3)	1244.95(17)	1245.15(11)	1247.31(16)
crystal size [mm^3]	0.420x0.259x0.136	0.330x0.255x0.178	0.330x0.255x0.178	0.4x0.3x0.2	0.4x0.3x0.2	0.4x0.3x0.2
Absorption coefficient [mm^{-1}]	5.923	3.129	3.135	3.134	3.133	2.463
F(000)	664	664	664	664	664	664
θ range [°] min.	2.958	2.329	2.333	2.331	2.332	2.587
max.	52.248	38.576	38.663	38.644	38.578	34.803
Resolution Range [Å] min.	6.886	6.901	6.889	6.895	6.892	5.689
max.	0.449	0.450	0.449	0.449	0.450	0.450
Collected ref.	124340	124178	96429	212039	125334	121728
Independent ref.	7162	7196	7123	7197	7171	7171
R_{int} [%]	4.57	2.46	3.23	2.21	2.59	3.51
Non-default SAINT input	PLANEBG = 1	SPOTSIZE = 0.8,<br>YSPOTSIZE = 0.8,<br>SPREAD = 0.5,<br>BGCORSCALE = -2	PROFXHALF = 12,<br>PROFYHALF = 12,<br>PROFZHALF = 12,<br>STRONGTHRESH = 15<br>LS_IOVS_MAX = 10	BGCORSCALE = -2		
R1(F) (all data, IAM) [%]	2.71	2.66	2.54	2.42	2.25	2.82

wR2(I) (all data, IAM) [%]	7.16	6.44	6.72	7.35	5.44	6.77
GOF (F^2, IAM)	1.075	1.065	1.110	1.098	1.102	1.115
Diff peak/hole (F^2, IAM) [eÅ^{-3}]	1.885	1.560	1.444	1.655	1.144	1.067
	-1.153	-0.769	-0.836	-0.383	-0.605	-0.644
Data in XD	7032	6719	6774	6952	6777	6660
Parameter in XD	162	162	162	162	162	162
R1(F) (all data, merged, XD) [%]	2.25	1.55	1.51	1.65	1.35	1.82
wR(I) (all data, merged, XD) [%]	2.30	1.27	1.49	1.89	1.28	1.42
GOF (XD)	4.901	2.027	1.528	5.636	2.123	1.676
Diff peak/hole (F^2, XD) [eÅ^{-3}]	1.603	0.508	0.392	0.992	0.371	0.574
	-0.626	-0.441	-0.248	-0.531	-0.401	-0.31
R1(F) (all data, merged, MoPro) [%]	2.24	1.54	1.49	1.58	1.33	1.80
wR2(I) (all data, merged, MoPro) [%]	2.24	1.29	1.46	1.84	1.24	1.39
GOF (MoPro)	4.799	2.059	1.504	5.517	2.056	1.646
Diff peak/hole (F^2, MoPro) [eÅ^{-3}]	1.719	0.579	0.438	1.206	0.442	0.712
	-0.778	-0.452	-0.344	-0.572	-0.512	-0.360
Data in MoPro	7032	6719	6774	6952	6777	6660
Parameter in MoPro	186	186	186	186	186	186

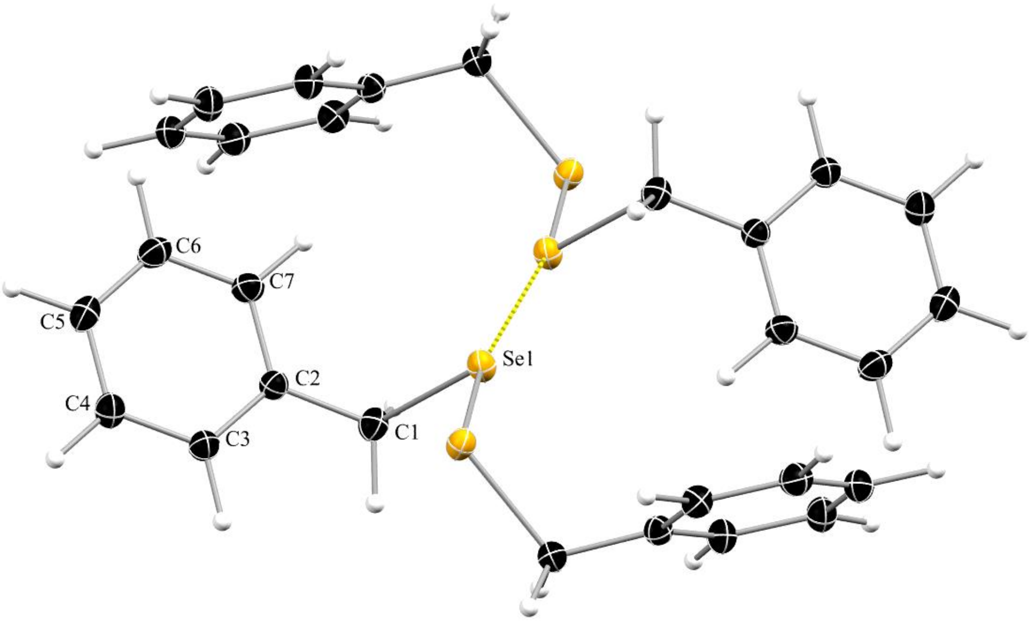

Fig. S1: Exemplary crystal structure of $(BnSe)_2$ from Dataset C. The structure contains half a molecule per asymmetric unit. Selected bond lengths [Å] and angles [°]: Se1-Se1 2.31475(11), Se1-C1 1.9833(4), intermolecular Se1-Se1 3.44249(11), C1-Se1-Se1 101.122(9), C1-Se1-Se1-C1 93.6(1)

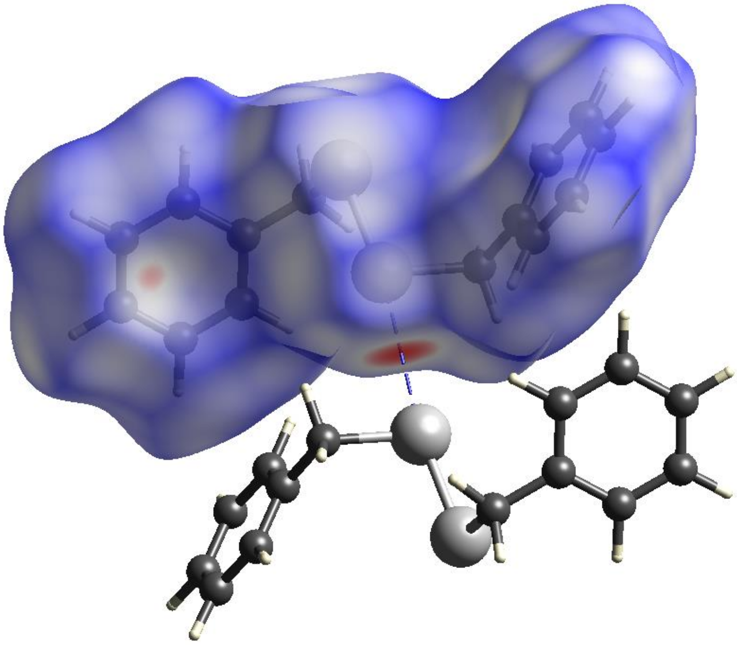

Fig. S2: Hirshfeld-Surface determined with CrystalExplorer17 [17].

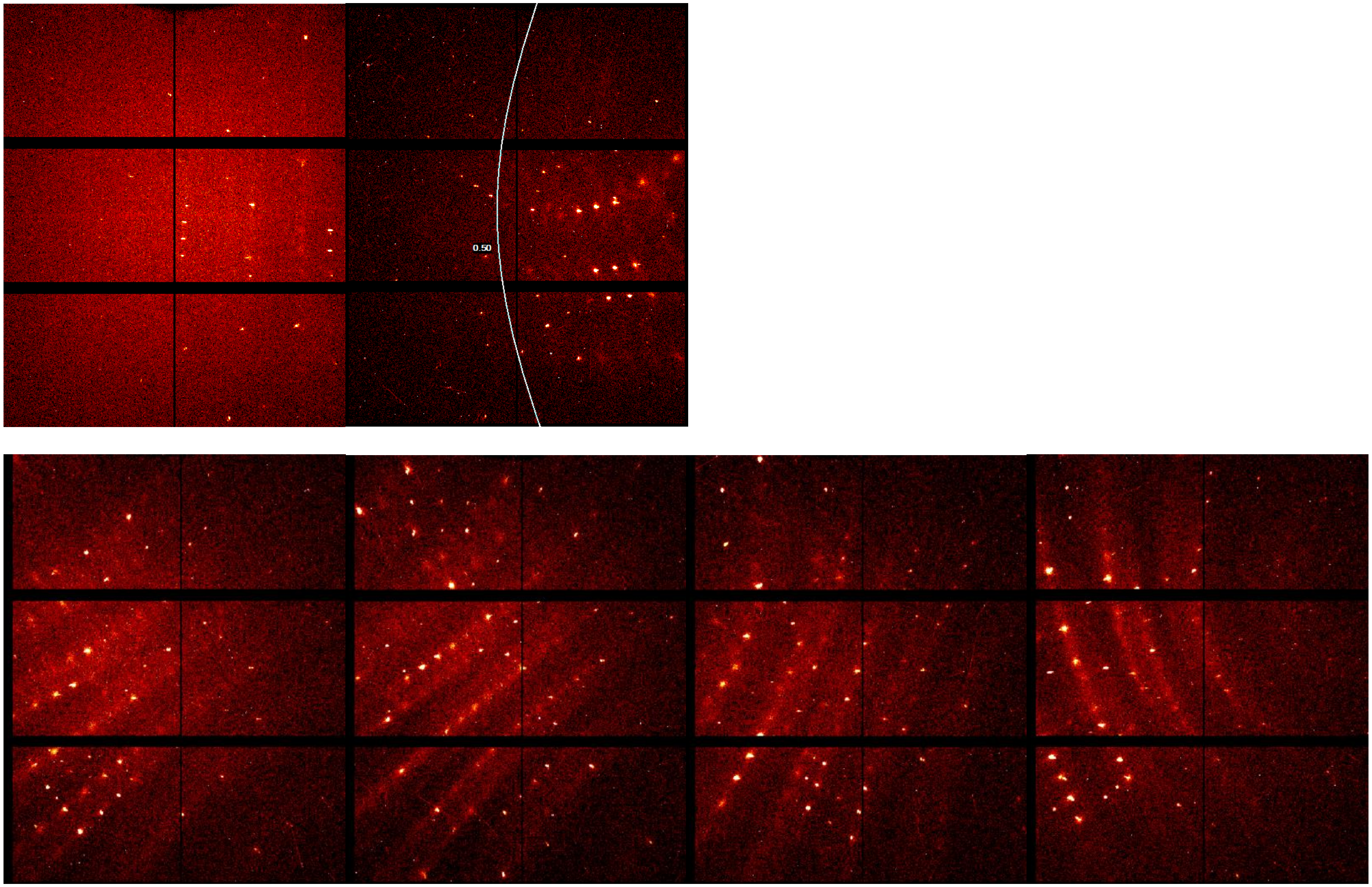

Fig. S3 Top: Frames of the Pilatus 3 300K CdTe pixel detector without and with adapted energy discrimination. Black pixel resemble an intensity of 0. Datasets **C** and **D** were collected with this detector. It provides a pixel wise adaptable energy-discrimination photon detection. By setting this threshold to a value above the selenium emission line at 12.7 KeV, the elevated background by X-ray fluorescence shown in the left image is effectively suppressed, leading to a very low background shown in the right image. The threshold was set to 15.5 keV for **C** and 13.0 keV for **D; Bottom:** four images with patterns that suggest diffuse scattering. Remarkably, this phenomenon occured right at the beginning of the data collection and did not aggravate.

Table S2.

Data quality statistics from XPREP of dataset **A**.

Resolution			Number of Data	Theory	Complete-ness [%]	Multiplicity	$\langle I \rangle$	$\langle I/\sigma \rangle$	R_{merge}	R_{sigma}	R_{rim}	R_{pim}
Inf	-	2	95	95	100	37.21	115.28	405.04	0.0537	0.0014	0.0546	0.0098
2	-	1.2	333	333	100	34.91	62.18	262.71	0.0411	0.002	0.0417	0.007
1.2	-	0.9	542	542	100	23.58	33.24	155.65	0.0438	0.0038	0.0448	0.0091
0.9	-	0.76	624	624	100	18.5	16.52	196.09	0.0584	0.0031	0.0601	0.0138
0.76	-	0.7	436	436	100	18.06	11.04	213.9	0.0401	0.0032	0.0412	0.0096
0.7	-	0.64	608	608	100	17.99	8.39	173.2	0.0317	0.0041	0.0326	0.0077
0.64	-	0.6	551	551	100	17.22	5.39	111.63	0.0347	0.0067	0.0358	0.0085
0.6	-	0.56	716	716	100	16.54	3.87	75.4	0.0392	0.0099	0.0404	0.0099
0.56	-	0.54	433	433	100	16.09	3.14	58.37	0.0443	0.013	0.0458	0.0114
0.54	-	0.52	513	513	100	15.37	2.53	46.85	0.0522	0.0167	0.054	0.0138
0.52	-	0.5	583	583	100	14.89	1.85	33.78	0.061	0.0232	0.0632	0.0163
0.5	-	0.49	357	357	100	13.62	1.59	28.85	0.0637	0.0282	0.0661	0.0176
0.49	-	0.48	356	356	100	12.68	1.27	23.42	0.0637	0.0345	0.0664	0.0183
0.48	-	0.47	390	390	100	12.03	1.21	20.63	0.0666	0.0384	0.0696	0.0199
0.47	-	0.46	441	441	100	11.84	1.19	20.76	0.0695	0.0384	0.0727	0.0211
0.46	-	0.45	473	473	100	10.74	0.97	17.21	0.0798	0.0493	0.0839	0.0253
0.45	-	0.45	16	25	64	1.84	0.56	5.5	0.1144	0.1815	0.1339	0.0679
0.55	-	0.45	3352	3361	99.7	13.24	1.63	29.49	0.0598	0.0275	0.0622	0.0167
Inf	-	0.45	7467	7476	99.9	17.07	10.96	101.76	0.0454	0.0049	0.0464	0.0092

Table S3.

Data quality statistics from XPREP of dataset **B**.

Resolution			Number of Data	Theory	Complete-ness [%]	Multiplicity	$\langle I \rangle$	$\langle I/\sigma \rangle$	R_{merge}	R_{sigma}	R_{rim}	R_{pim}
Inf	-	2	95	95	100	20.37	137.66	285.6	0.0123	0.0018	0.0126	0.003
2	-	1.2	334	334	100	23.23	74.76	194.7	0.0182	0.0027	0.0186	0.004
1.2	-	0.9	544	544	100	26.27	39.1	206.12	0.0229	0.0027	0.0234	0.0047
0.9	-	0.76	627	627	100	24.62	19.77	157.92	0.0237	0.0035	0.0242	0.005
0.76	-	0.7	434	434	100	20.49	13.13	112.81	0.0265	0.0054	0.0272	0.0061
0.7	-	0.64	616	616	100	19.31	10	87.67	0.0302	0.0076	0.0311	0.0071
0.64	-	0.6	555	555	100	17.88	6.68	60.97	0.0378	0.0117	0.039	0.0093
0.6	-	0.56	717	717	100	16.1	4.78	42.21	0.0427	0.0172	0.0442	0.011
0.56	-	0.54	438	438	100	14.5	3.88	34.51	0.0426	0.0228	0.0442	0.0114
0.54	-	0.52	512	512	100	13.88	3.11	27.24	0.0492	0.0298	0.0511	0.0135
0.52	-	0.5	585	585	100	13.59	2.33	20.21	0.0587	0.0416	0.0611	0.0164
0.5	-	0.49	358	358	100	13.11	2.01	16.82	0.0658	0.0505	0.0686	0.0187
0.49	-	0.48	352	352	100	12.5	1.67	13.86	0.0739	0.063	0.0772	0.0216
0.48	-	0.47	392	392	100	12.66	1.61	13.4	0.077	0.0671	0.0803	0.0221
0.47	-	0.46	444	444	100	12.06	1.52	12.35	0.0794	0.0748	0.083	0.0233
0.46	-	0.45	472	472	100	11.55	1.21	9.55	0.0917	0.0964	0.0961	0.0278
0.45	-	0.45	27	29	93.1	4.1	2.05	8.76	0.0614	0.1172	0.0694	0.0305
0.55	-	0.45	3373	3375	99.9	12.87	2.06	17.5	0.0626	0.0506	0.0652	0.0178
Inf	-	0.45	7502	7504	100	17.07	13.14	71.66	0.0244	0.0075	0.0251	0.0056

Table S4.

Data quality statistics from XPREP of dataset **C**.

Resolution			Number of Data	Theory	Complete-ness [%]	Multiplicity	$\langle I \rangle$	$\langle I/\sigma \rangle$	R_{merge}	R_{sigma}	R_{rim}	R_{pim}
Inf	-	2	95	95	100	14.42	136.87	196.31	0.0172	0.003	0.0179	0.0049
2	-	1.2	334	334	100	18	73.59	153.5	0.0221	0.0036	0.0228	0.0055
1.2	-	0.9	542	542	100	23.29	38.38	136.28	0.0278	0.0038	0.0285	0.0059
0.9	-	0.76	626	626	100	22.51	19.17	91.7	0.0338	0.0056	0.0346	0.0072
0.76	-	0.7	435	435	100	18.69	12.83	69.78	0.0381	0.0078	0.0392	0.0089
0.7	-	0.64	607	611	99.3	14.69	9.91	55.35	0.042	0.0105	0.0435	0.0111
0.64	-	0.6	551	555	99.3	12.59	6.48	40.89	0.0476	0.0147	0.0496	0.0138
0.6	-	0.56	707	717	98.6	11.9	4.72	30.18	0.0603	0.0192	0.063	0.018
0.56	-	0.54	434	437	99.3	8.8	3.79	21.99	0.0696	0.0284	0.0737	0.0236
0.54	-	0.52	502	512	98	9.29	3.18	19.43	0.0794	0.032	0.0839	0.0265
0.52	-	0.5	575	583	98.6	9.69	2.34	15.66	0.0983	0.0392	0.1037	0.0326
0.5	-	0.49	347	352	98.6	9.39	2	13.8	0.1057	0.0448	0.1115	0.0352
0.49	-	0.48	347	354	98	9.12	1.66	12.22	0.1176	0.0524	0.1247	0.0408
0.48	-	0.47	395	399	99	9.37	1.6	12.06	0.1149	0.053	0.1215	0.0391
0.47	-	0.46	433	442	98	8.79	1.53	11.65	0.1248	0.0567	0.1322	0.0431
0.46	-	0.45	461	469	98.3	8.17	1.22	8.89	0.1393	0.0725	0.1484	0.0502
0.45	-	0.45	29	45	64.4	1.02	1.61	5.21	0.0897	0.1595	0.1231	0.0839
0.55	-	0.45	3315	3385	97.9	8.91	2.06	13.82	0.1009	0.0453	0.1069	0.0346
Inf	-	0.45	7420	7508	98.8	13.19	13.03	48.57	0.0323	0.0086	0.0333	0.0081

Table S5.

Data quality statistics from XPREP of dataset **D**.

Resolution			Number of Data	Theory	Complete-ness [%]	Multiplicity	$\langle I \rangle$	$\langle I/\sigma \rangle$	R_{merge}	R_{sigma}	R_{rim}	R_{pim}
Inf	-	2	94	95	98.9	21.67	157.86	808.65	0.0049	0.0007	0.005	0.0013
2	-	1.2	334	334	100	28.51	88.66	568.52	0.0079	0.0009	0.0081	0.0017
1.2	-	0.9	545	545	100	31.3	45.31	355.89	0.0126	0.0015	0.0128	0.0025
0.9	-	0.76	629	629	100	32.86	22.23	221.1	0.0184	0.0024	0.0188	0.0035
0.76	-	0.7	431	431	100	27.49	14.47	154.47	0.0257	0.0036	0.0262	0.0053
0.7	-	0.64	618	618	100	31.5	10.94	134.05	0.0299	0.0044	0.0305	0.0056
0.64	-	0.6	557	557	100	31.39	7.2	98.62	0.0383	0.0064	0.039	0.0071
0.6	-	0.56	713	713	100	31.12	5.07	70.6	0.0456	0.0088	0.0464	0.0084
0.56	-	0.54	440	440	100	30.79	4.14	58.96	0.0512	0.011	0.0521	0.0094
0.54	-	0.52	515	515	100	30.03	3.34	48.65	0.0578	0.0138	0.0588	0.0107
0.52	-	0.5	585	585	100	28.86	2.45	36.21	0.0658	0.0191	0.067	0.0124
0.5	-	0.49	363	363	100	27.02	2.11	30.74	0.073	0.0232	0.0744	0.0141
0.49	-	0.48	350	350	100	26.88	1.76	25.66	0.0831	0.0281	0.0847	0.0163
0.48	-	0.47	391	391	100	25.49	1.68	23.18	0.0869	0.0316	0.0887	0.0175
0.47	-	0.46	445	445	100	24.39	1.58	21.36	0.092	0.035	0.094	0.019
0.46	-	0.45	471	471	100	23.42	1.34	17.48	0.105	0.0428	0.1074	0.0221
0.45	-	0.45	22	25	88	4.36	2.18	11.62	0.0939	0.0865	0.1024	0.0383
0.55	-	0.45	3375	3378	99.9	26.83	2.18	31.19	0.0713	0.0235	0.0727	0.0139
Inf	-	0.45	7503	7507	99.9	28.96	14.95	129.68	0.0219	0.0036	0.0223	0.0043

Table S6.

Data quality statistics from XPREP of dataset **E**.

Resolution			Number of Data	Theory	Complete-ness [%]	Multiplicity	$\langle I \rangle$	$\langle I/\sigma \rangle$	R_{merge}	R_{sigma}	R_{rim}	R_{pim}
Inf	-	2	92	95	96.8	11.49	132.82	161.72	0.0143	0.0052	0.0157	0.0063
2	-	1.2	334	334	100	20.04	74.76	202.35	0.0193	0.0039	0.0204	0.0063
1.2	-	0.9	544	544	100	24.57	39.96	219.61	0.0216	0.0032	0.0223	0.0053
0.9	-	0.76	625	625	100	25.69	20.31	191.71	0.0222	0.0028	0.0227	0.0045
0.76	-	0.7	434	434	100	22.63	13.69	139.98	0.0252	0.0041	0.0258	0.0054
0.7	-	0.64	615	615	100	19.92	10.49	108.14	0.0264	0.0055	0.0271	0.006
0.64	-	0.6	552	552	100	17.73	7.03	76.86	0.03	0.0083	0.0309	0.0073
0.6	-	0.56	717	717	100	16.58	5.14	54.99	0.0348	0.0115	0.0359	0.0088
0.56	-	0.54	433	433	100	15.96	4.16	44.21	0.0404	0.0147	0.0418	0.0104
0.54	-	0.52	513	513	100	14.78	3.42	35.96	0.0459	0.0186	0.0476	0.0123
0.52	-	0.5	591	591	100	13.85	2.59	27.06	0.055	0.0253	0.0571	0.0153
0.5	-	0.49	359	359	100	13.01	2.16	22.17	0.0611	0.0312	0.0636	0.0175
0.49	-	0.48	346	346	100	12.75	1.82	19.1	0.0681	0.0375	0.0709	0.0197
0.48	-	0.47	398	398	100	12.42	1.77	17.97	0.0705	0.0401	0.0736	0.0209
0.47	-	0.46	443	443	100	12.31	1.69	17.27	0.0764	0.0429	0.0797	0.0225
0.46	-	0.45	468	470	99.6	11.47	1.39	13.66	0.0891	0.0541	0.0932	0.0271
0.45	-	0.45	8	23	34.8	1.13	1.8	7.93	0.1389	0.108	0.163	0.0848
0.55	-	0.45	3353	3370	99.5	13.13	2.27	23.61	0.0586	0.0302	0.061	0.0166
Inf	-	0.45	7472	7492	99.7	17.15	13.41	83.01	0.0256	0.0064	0.0265	0.0065

Table S7.

Data quality statistics from XPREP of dataset **F**.

Resolution			Number of Data	Theory	Complete-ness [%]	Multiplicity	$\langle I \rangle$	$\langle I/\sigma \rangle$	R_{merge}	R_{sigma}	R_{rim}	R_{pim}
Inf	-	2	90	95	94.7	14.45	124.61	160.41	0.0244	0.0049	0.0261	0.0086
2	-	1.2	334	334	100	21.63	64.77	201.11	0.0233	0.0033	0.0241	0.006
1.2	-	0.9	545	545	100	23.74	33.79	166.02	0.0241	0.0032	0.0246	0.0051
0.9	-	0.76	627	627	100	22.88	16.74	98.86	0.0302	0.0053	0.0309	0.0064
0.76	-	0.7	434	434	100	21.6	11.31	74.04	0.0353	0.0077	0.0362	0.0077
0.7	-	0.64	615	615	100	20.03	8.37	56.18	0.0416	0.0105	0.0427	0.0095
0.64	-	0.6	557	557	100	18.48	5.55	38.85	0.0553	0.016	0.0568	0.0131
0.6	-	0.56	714	714	100	16.66	3.96	26.8	0.0669	0.0227	0.0691	0.0169
0.56	-	0.54	439	439	100	14.97	3.23	21.94	0.0744	0.029	0.077	0.0197
0.54	-	0.52	517	517	100	13.91	2.57	17.33	0.0838	0.0372	0.087	0.0232
0.52	-	0.5	582	582	100	13.09	1.9	12.83	0.1062	0.0509	0.1106	0.0305
0.5	-	0.49	362	362	100	12.47	1.64	11.16	0.1173	0.06	0.1224	0.0345
0.49	-	0.48	353	353	100	12.14	1.35	9.42	0.1386	0.0723	0.1447	0.0414
0.48	-	0.47	390	390	100	11.83	1.28	8.7	0.1504	0.0801	0.1573	0.0458
0.47	-	0.46	445	445	100	11.68	1.2	8.31	0.1592	0.0857	0.1666	0.0488
0.46	-	0.45	471	472	99.8	10.65	0.99	6.61	0.1806	0.1111	0.1897	0.0577
0.45	-	0.45	2	3	66.7	1.33	3.25	7.21	0.0724	0.0978	0.1024	0.0724
0.55	-	0.45	3354	3356	99.9	12.47	1.68	11.44	0.1145	0.0595	0.1193	0.0335
Inf	-	0.45	7477	7484	99.9	16.67	11.26	51.51	0.0346	0.0095	0.0356	0.0085

Table S8.

Local coordinate system for the charge density refinement in XD and MoPro.

ATOM	ATOM0	AX1	ATOM1	ATOM2	AX2	GC-level	κ-set	SITESYM	CHEMCON
Se(1)	DUM0	Z	Se(1)	C(1)	Y	4	1	_mZ → NO	
C(1)	Se(1)	Z	C(1)	C(2)	Y	2	2	_mZ	
C(2)	C(1)	Z	C(2)	C(3)	Y	2	3	_mXmY2Z	
C(3)	C(6)	Z	C(3)	C(2)	Y	2	3	_mXmY2Z	
C(4)	C(7)	Z	C(4)	C(3)	Y	2	3	_mXmY2Z	C(3)
C(5)	C(2)	Z	C(5)	C(4)	Y	2	3	_mXmY2Z	C(3)
C(6)	C(3)	Z	C(6)	C(7)	Y	2	3	_mXmY2Z	C(3)
C(7)	C(4)	Z	C(7)	C(2)	Y	2	3	_mXmY2Z	C(3)
H(1A)	C(1)	Z	H(1A)	H(1B)	Y	1	4	_cy	
H(1B)	C(1)	Z	H(1B)	H(1A)	Y	1	4	_cy	H(1A)
H(3)	C(3)	Z	H(3)	C(2)	Y	1	4	_cy	
H(4)	C(4)	Z	H(4)	C(5)	Y	1	4	_cy	H(3)
H(5)	C(5)	Z	H(5)	C(4)	Y	1	4	_cy	H(3)
H(6)	C(6)	Z	H(6)	C(7)	Y	1	4	_cy	H(3)
H(7)	C(7)	Z	H(7)	C(6)	Y	1	4	_cy	H(3)

Table S9. XD refinement strategy, exemplary for dataset **C**. Abbreviations: MP: Multipole, M: monopoles; D: dipoles; Q: quadrupoles; O: octupoles; H: hexadecapoles, K: κ, U2, U3, U4: Gram Charlier 2nd, 3rd and 4th order, HXYZ: hydrogen position against data up to 0.5 sin(θ)/λ.

Step	New Parameter	D < 0.5 sin(th)/l	#MP-Param	Data	Para	Data/Para	$R(F^2)$	$wR(F^2)$	GOF
1	SCALE	644	0	6774	1	6774	0.0427	0.0515	5.231
2	DQOH	644	46	6774	47	144.1	0.0370	0.0395	4.023
3	U2	644	46	6774	95	71.3	0.0233	0.0264	2.704
4	κ	644	49	6774	98	69.1	0.0151	0.0192	1.966
5	XYZ	644	49	6774	122	55.5	0.0147	0.0184	1.889
6	M	644	55	6774	127	53.3	0.0141	0.0178	1.823
7	H-XYZ	644	0	644	22	29.3	0.0119	0.0167	3.974
8	all prior	644	55	6774	127	53.3	0.0139	0.0172	1.767
9	U3(Se)	644	0	6774	11	615.8	0.0138	0.0171	1.736
10	all prior + U3(Se)	644	55	6774	137	49.5	0.0137	0.0170	1.740
11	U4(Se)	644	0	6774	16	423.4	0.0133	0.0167	1.698
12	all prior + U4(Se)	644	55	6774	152	44.6	0.0127	0.0157	1.609
13	Se NoSymm	644	65	6774	162	41.8	0.0124	0.0153	1.569
14	κ'	644	3	6774	4	1693.5	0.0123	0.0151	1.530
15	all prior	644	65	6774	162	41.8	0.0121	0.0149	1.528

Parameters were added stepwise to the refinement. Starting with multipole parameters, anisotropic motion and positional parameters, the refinement was extended by the application of anharmonic motion and the refinement of all selenium multipole parameters. In the penultimate step, κ' parameters were refined, followed by a refinement of all prior introduced parameters. Hydrogen positional parameter were refined only against low-resolution data up to 0.5 sin(θ)/λ, while constrained to neutron distances.

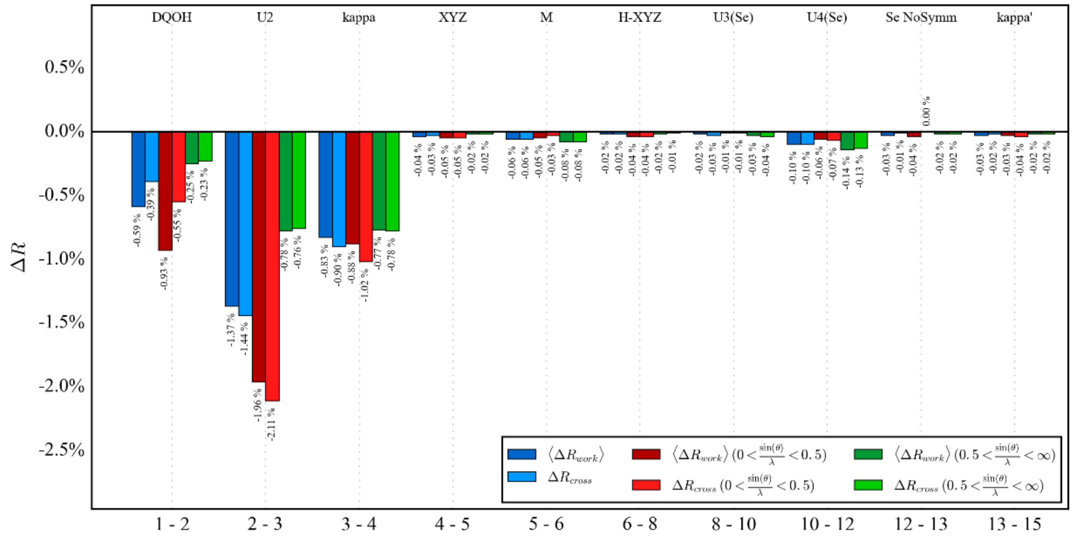

Fig. S4: ΔR_{cross} values of the XD refinement exemplary for dataset **C** to check for overfitting [13] . Abbreviations: M: monopoles; D: dipoles; Q: quadrupoles; O: octupoles; H: hexadecapoles, U2, U3, U4: Gram Charlier 2nd, 3rd and 4th order, HXYZ: hydrogen position against data up to 0.5 sin(θ)/λ.

Table S10.

MoPro refinement strategy, exemplary for dataset **C**. Abbreviations: M: monopoles; D: dipoles; Q: quadrupoles; O: octupoles; H: hexadecapoles, K: κ, U2, U3, U4: Gram Charlier 2nd, 3rd and 4th order, HXYZ: hydrogen position against data up to 0.5 sin(θ)/λ.

step	New Parameter	D < 0.5 sin(th)/l	#MP-Param	Data	Para	Data/Para	$R(F^2)$	$wR(F^2)$	GOF
1	SCALE	644	0	6774	1	6770.0	3.736	4.751	4.936
2	DQOH	644	46	6774	47	144.0	3.156	3.517	3.631
3	U2	644	46	6774	95	71.3	2.330	2.679	2.783
4	κ	644	49	6774	98	69.1	1.516	1.914	1.990
5	XYZ	644	49	6774	122	55.5	1.463	1.816	1.889
6	M	644	56	6774	127	53.3	1.409	1.759	1.831
7	H-XYZ	644	0	644	22	29.3	1.223	1.673	4.050
8	all prior	644	56	6774	127	53.3	1.388	1.713	1.783
9	U3 + U4	644	56	6774	152	44.6	1.287	1.573	1.639
10	Se NoSymm	644	56	6774	162	41.8	1.252	1.534	1.600
11	κ'	644	3	6774	4	1690.0	1.247	1.514	1.560
12	all prior	644	56	6774	162	41.8	1.222	1.495	1.559
13	32P (Se)	644	67	6774	173	39.2	1.215	1.484	1.549
14	64P (Se)	644	80	6774	186	36.4	1.197	1.461	1.527

The refinement strategy is analogous to the refinement strategy in XD (Table S9), but slightly shortened due to program restrictions. Additionally, 32-poles at selenium were introduced in the penultimate and 64-poles in the ultimate step of the refinement.

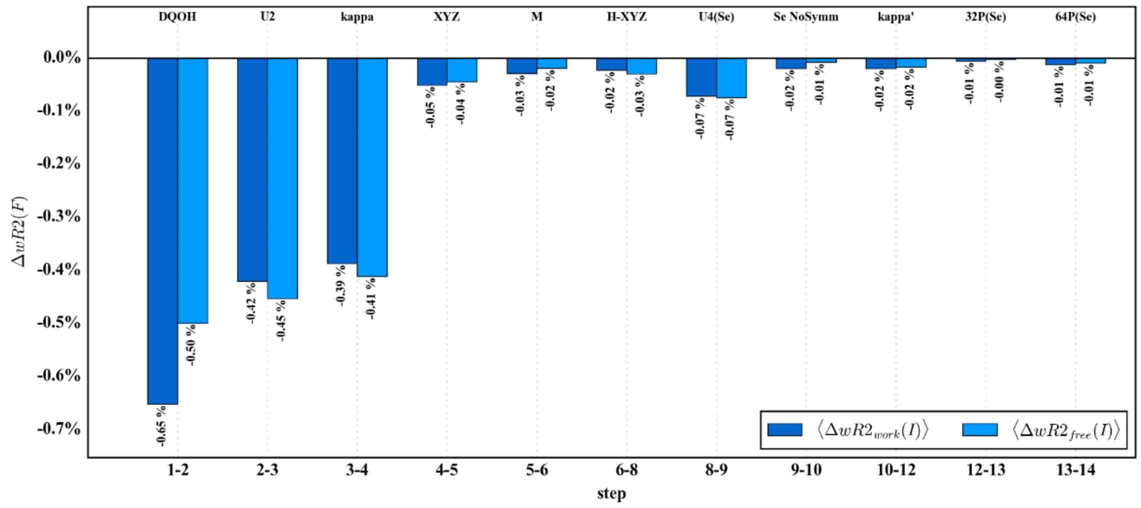

Fig. S5: $\langle \boldsymbol{R_{free}} \rangle$ values for the Mopro refinement of dataset **C**. Abbreviations: M: monopoles; D: dipoles; Q: quadrupoles; O: octupoles; H: hexadecapoles, U2, U3, U4: Gram Charlier 2nd, 3rd and 4th order, HXYZ: hydrogen position against data up to 0.5 sin(θ)/λ.

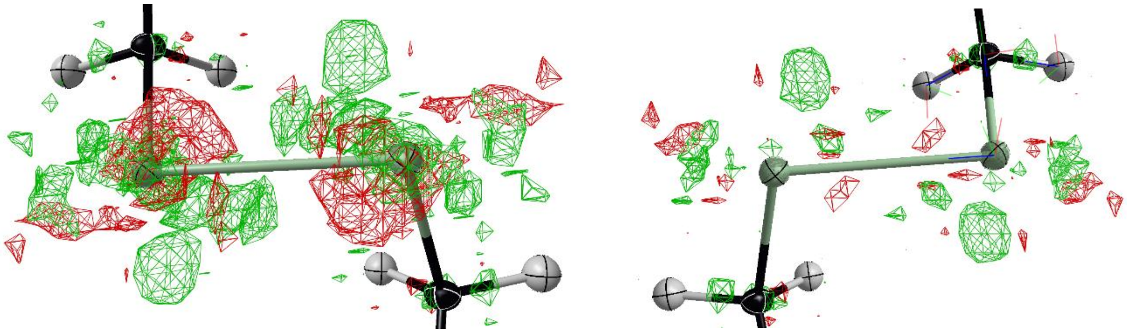

Fig. S6: The isosurface value is ±0.15 eÅ^{-3}. Positive contours are plotted with green lines and negative contours are plotted with red lines. The graphics were created with MoleCoolQT [18]

Table S11.

Consistency check for the refinement of Gram Charlier parameters of 3rd and 4th order at Se(1) in XD. Analysis of the Probability Density Function (PDF). Extreme displacements in the map from the equilibrium position. ΔX/Y/Z = -0.80 to 0.80Å. For datasets **B** to **F**, the minimum PDF value and integrated volume for negative probability are reasonably low, while for dataset **A**, the values are slightly elevated. In order to retain comparability, anharmonic motion was refined anyway.

Set	Minimum PDF value	Maximum PDF Value	Total integrated negative probability [%]	Integrated volume for negative probability [Å^{-3}]	Total integrated positive probability [%]	Integrated volume for positive probability [Å^{-3}]
**A**	-159.77	53291.07	-0.23	1.79	100.23	2.50
**B**	-33.65	60841.29	-0.02	0.58	100.02	3.72
**C**	0.00	60694.31	0.00	0.00	100.00	4.29
**D**	0.00	57001.57	0.00	0.00	100.00	4.29
**E**	-6.81	63790.20	0.00	0.57	100.00	3.72
**F**	-15.72	55020.99	-0.01	0.68	100.01	3.62

Table S12.

For an anharmonic refinement, Kuhs's rule [12] should be fulfilled. However, this rule seems to be too strict for heavier atoms. [19] Therefore, anharmonic motion of Se(1) was refined for all datasets up to the fourth order although the used resolution is only $\sin(\theta)/\lambda = 1.12$ Å^{-1}.

Dataset	Principal M.D.A's (Å)			Min. resolution [Å^{-1}]	
				**n = 3**	**n = 4**
**A**	0.129	0.118	0.096	1.14	1.32
**B**	0.136	0.118	0.095	1.12	1.30
**C**	0.137	0.118	0.096	1.12	1.29
**D**	0.145	0.124	0.104	1.05	1.22
**E**	0.132	0.111	0.092	1.17	1.35
**F**	0.134	0.118	0.097	1.12	1.30

Table S13.

Significance check for the refinement of Gram Charlier parameters of 3rd and 4th order at Se(1) in XD. Gram-Charlier parameter divided by their error – in order to be significant, values need to be larger than 3.

Dataset	C111/σ	C222/σ	C333/σ	C112/σ	C122/σ	C113/σ	C133/σ	C223/σ	C233/σ	C123/σ
	D1111/σ	D2222/σ	D3333/σ	D1112/σ	D1222/σ	D1113/σ	D1333/σ	D2223/σ	D2333/σ	D1122/σ
	D1133/σ	D2233/σ	D1123/σ	D1223/σ	D1233/σ					
**A**	3.8	7.1	0.5	0.1	4.9	1.5	2.7	0.4	2.4	2.0
	3.0	11.1	14.3	4.0	4.4	3.0	3.0	2.7	3.8	5.5
	6.0	5.3	2.5	29.0	2.5					
**B**	13.0	2.2	13.5	0.8	17.4	21.0	15.5	8.2	1.0	2.0
	2.0	10.0	44.5	7.5	3.4	inf	4.0	4.3	8.0	21.0
	2.0	18.5	7.0	9.0	5.5					
**C**	4.7	5	1.8	2	10.8	4.5	5.5	2.6	4.8	3
	12	24.46	17	3	2.6	8	14	0.2	0.5	10
	11	15	3	8	5					
**D**	9.5	1.43	3.43	0.67	6.75	5.67	7	2.89	2.44	0.5
	12.5	21.61	25.25	4.5	5.11	4	14	4.82	7	11
	17	21.25	4.5	4.5	9					
**E**	11.0	4.0	6.3	1.0	15.8	12.0	17.0	3.4	1.0	2.0
	1.0	22.7	12.0	5.0	5.0	inf	0.0	2.2	5.0	6.0
	3.0	4.0	3.0	5.0	8.0					
**F**	9.0	4.2	0.8	0.2	11.0	5.0	7.5	1.5	1.7	1.5
	9.0	2.6	11.7	2.5	5.2	1.0	6.0	2.0	3.7	1.5
	4.0	3.0	2.0	1.0	4.5					

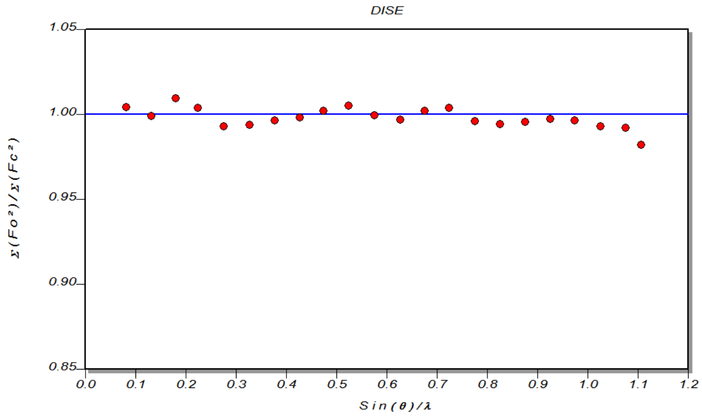

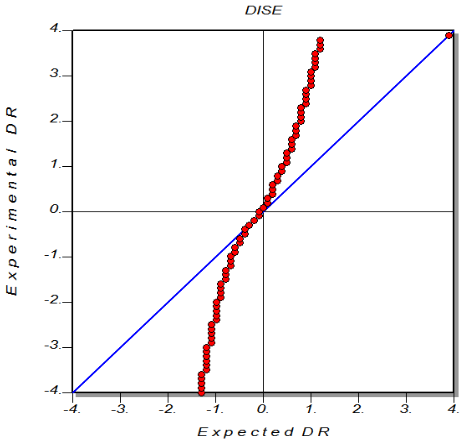

Fig. S7:
DRK-Plot [13–15] for the XD refinement of dataset **A**.

Fig. S8:
Normal Probability Plot $DR = (F_o^2 - F_c^2)/\sigma^2)$ ([20] for the XD refinement of dataset **A.**

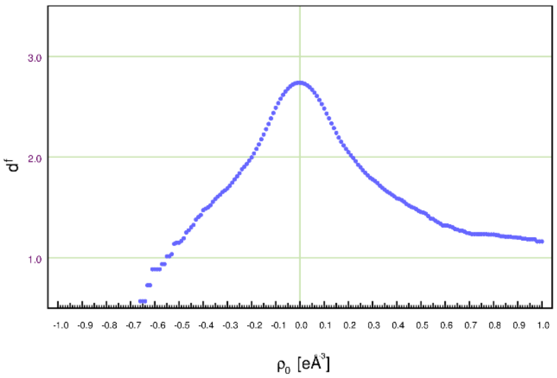

Fig. S9:
Fractal dimension plot [16] for the XD refinement of dataset **A**.

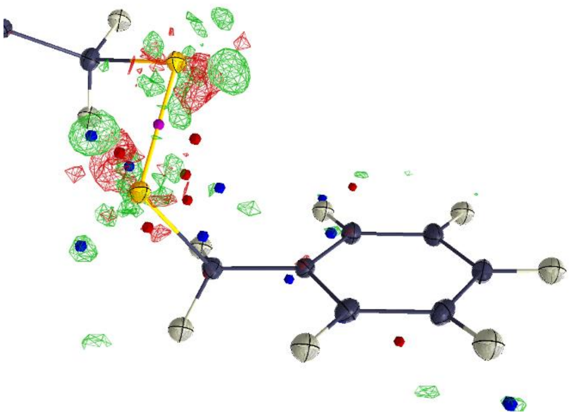

Fig. S10:
Residual density plot for the XD refinement of dataset **A**. +0.3eÅ^{-1} green, -0.3eÅ^{-1} red, maxima blue, minima red.

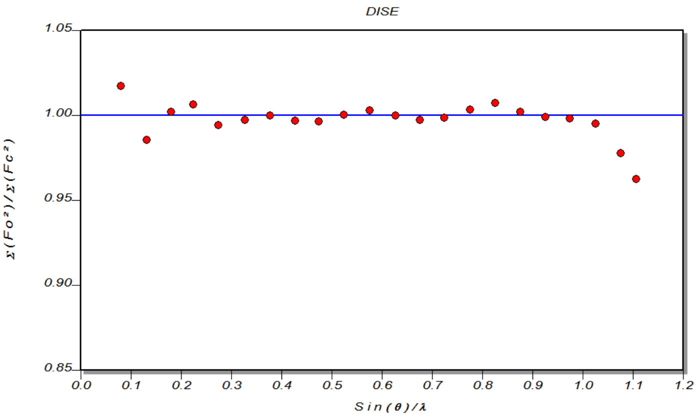

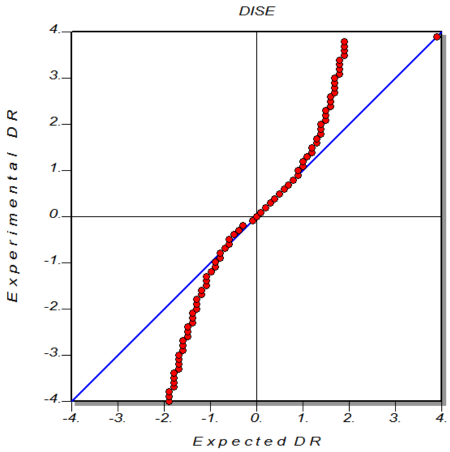

Fig. S11:
DRK-Plot [13–15] for the XD refinement of dataset **B**.

Fig. S12:
Normal Probability Plot $DR = (F_o^2 - F_c^2)/\sigma^2)$ ([20] for the XD refinement of dataset **B.**

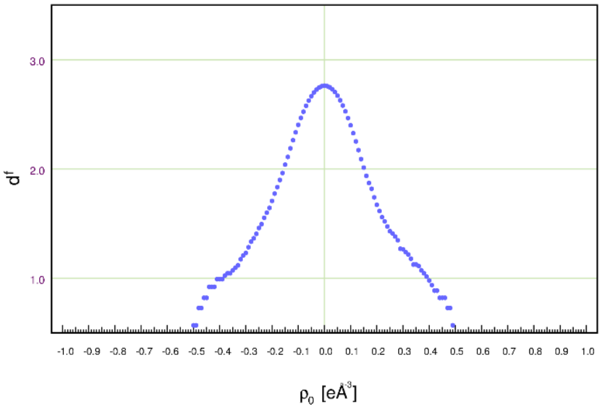

Fig. S13:
Fractal dimension plot [16] for the XD refinement of dataset **B**.

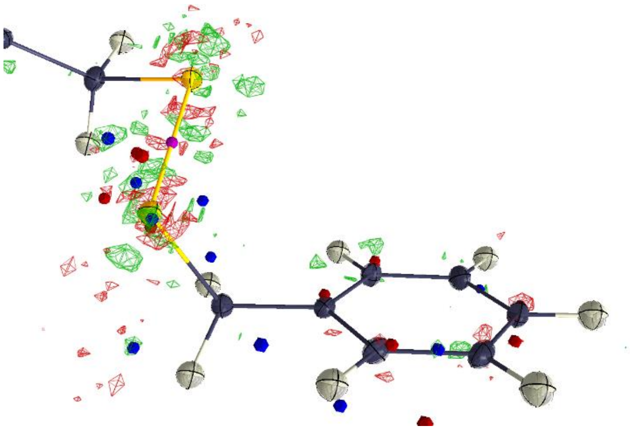

Fig. S14:
Residual density plot for the XD refinement of dataset **B**. Isolevels: +0.16 eÅ^{-1} green, -0.16 eÅ^{-1} red; maxima blue, minima red.

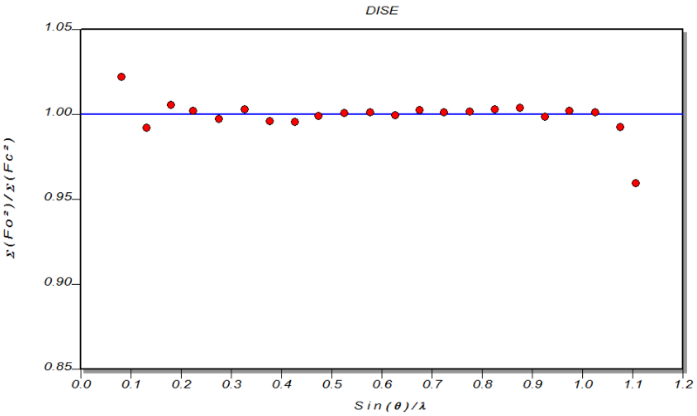

Fig. S15:
DRK-Plot [13–15] for the XD refinement of dataset **C**.

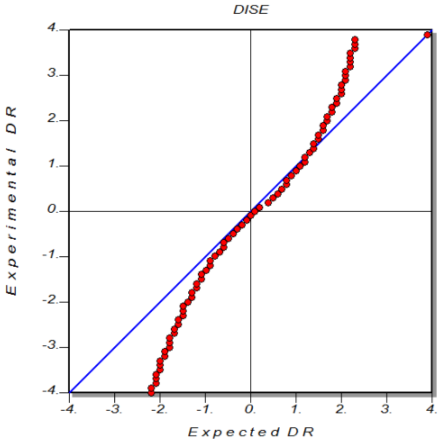

Fig. S16:
Normal Probability Plot $DR = (F_o^2 - F_c^2)/\sigma^2)$ ([20] for the XD refinement of dataset **C.**

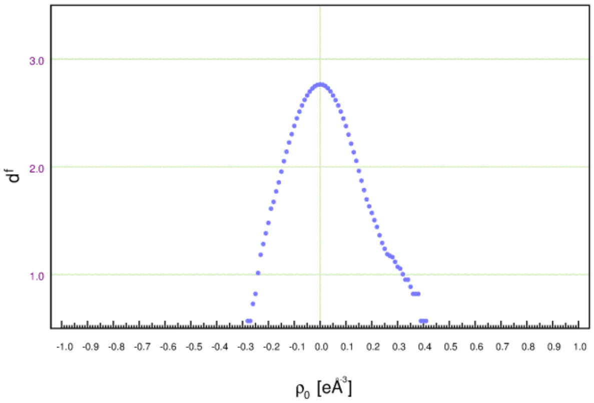

Fig. S17:
Fractal dimension plot [16] for the XD refinement of dataset **C**.

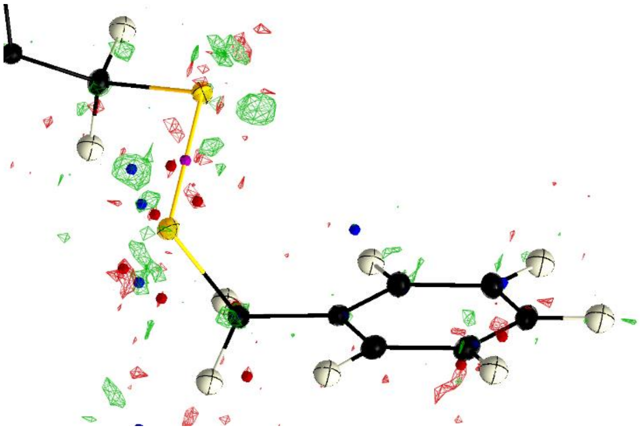

Fig. S18:
Residual density plot for the XD refinement of dataset **C**. Isolevels: +0.15 eÅ^{-1} green, -0.15 eÅ^{-1} red; maxima blue, minima red.

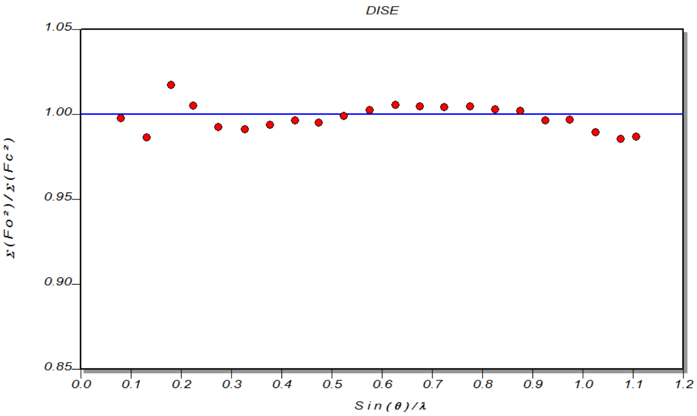

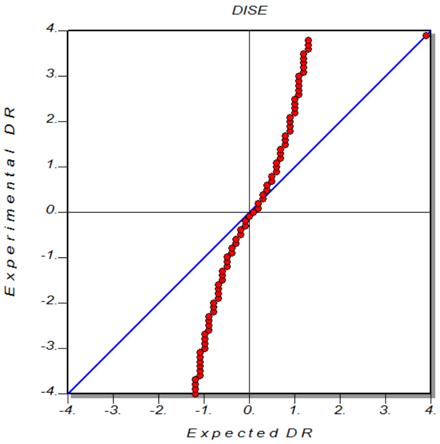

Fig. S19:
DRK-Plot [13–15] for the XD refinement of dataset **D**.

Fig. S20:
Normal Probability Plot $DR = (F_o^2 - F_c^2)/\sigma^2)$ ([20] for the XD refinement of dataset **D.**

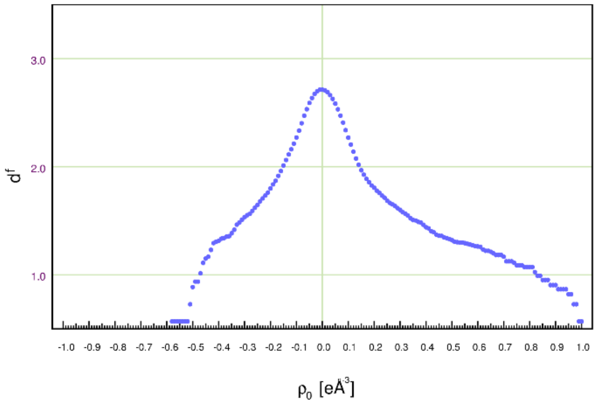

Fig. S21:
Fractal dimension plot [16] for the XD refinement of dataset **D**.

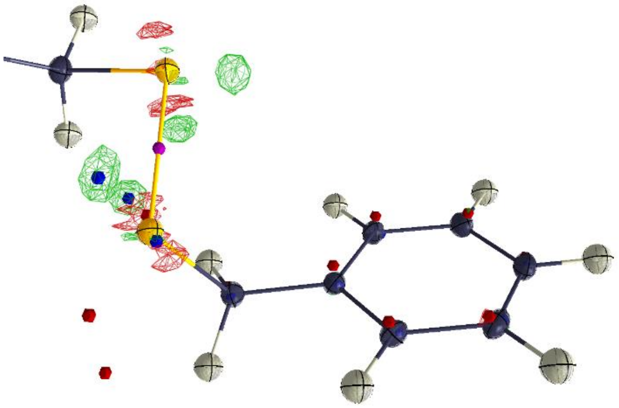

Fig. S22:
Residual density plot for the XD refinement of dataset **D**. Isolevels: +0.3 eÅ^{-1} green, -0.3 eÅ^{-1} red; maxima blue, minima red.

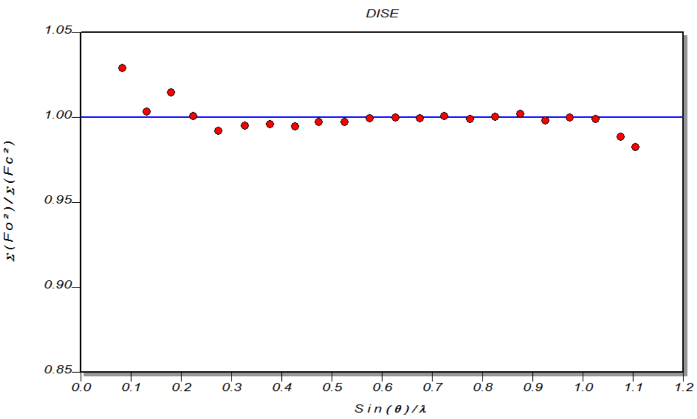

Fig. S23:
DRK-Plot [13–15] for the XD refinement of dataset **E**.

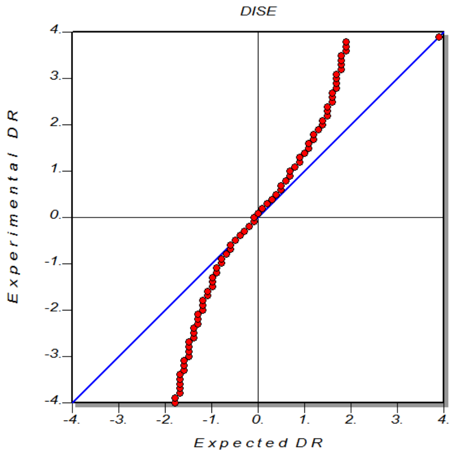

Fig. S24:
Normal Probability Plot $DR = (F_o^2 - F_c^2)/\sigma^2)$ ([20] for the XD refinement of dataset **E.**

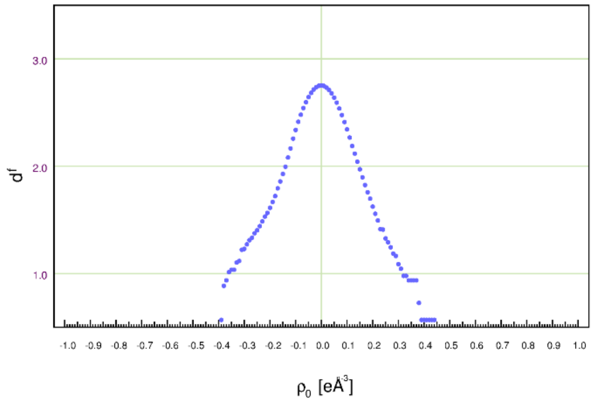

Fig. S25:
Fractal dimension plot [16] for the XD refinement of dataset **E**.

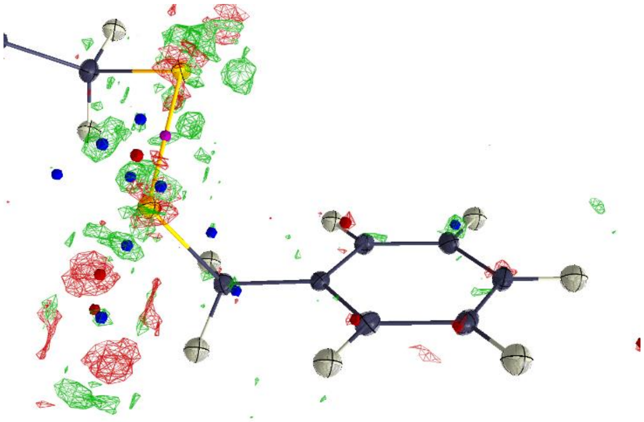

Fig. S26:
Residual density plot for the XD refinement of dataset **E**. Isolevels: +0.15 eÅ^{-1} green, -0.15 eÅ^{-1} red; maxima blue, minima red.

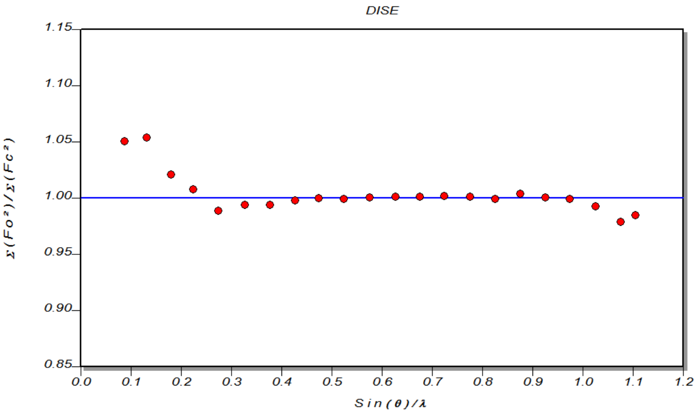

Fig. S27:
DRK-Plot [13–15] for the XD refinement of dataset **F**.

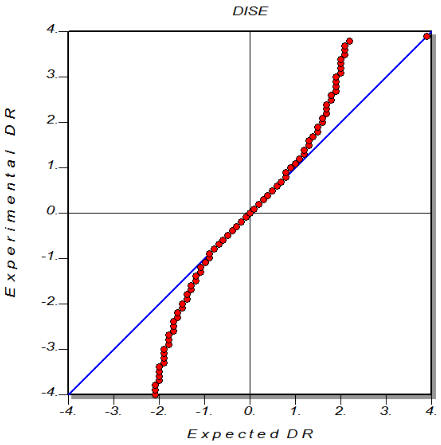

Fig. S28:
Normal Probability Plot $DR = (F_o^2 - F_c^2)/\sigma^2)$ ([20] for the XD refinement of dataset **F.**

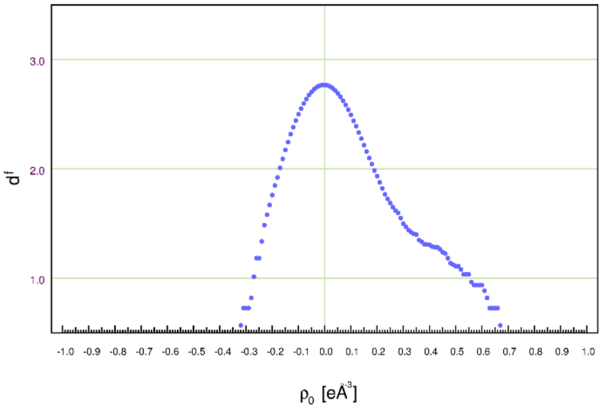

Fig. S29:
Fractal dimension plot [16] for the XD refinement of dataset **F**.

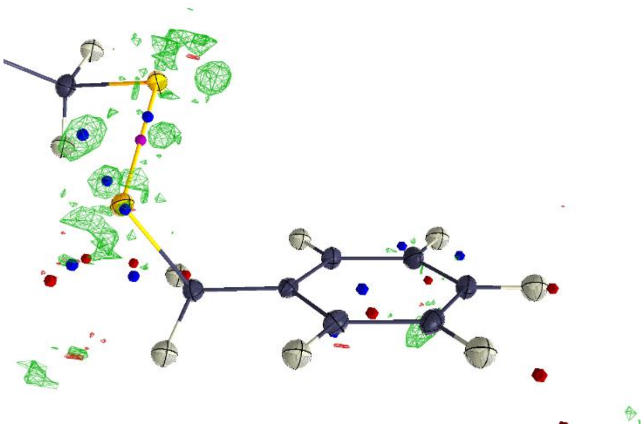

Fig. S30:
Residual density plot for the XD refinement of dataset **F**. Isolevels: +0.2 eÅ^{-1} green, -0.2 eÅ^{-1} red; maxima blue, minima red.

Computational Details

All calculations were carried out with the additive crystal QM/MM model (AC-QM/MM)[21] implemented in a modified version of the Chemshell 3.5.0 [22] program. The built-in DL-POLY module was used for the MM part, the ORCA 3.0.3 [23] program package was used for the QM part. QM calculations employed the B3LYP functional [24] in conjunction with the def2-TZVP basis set [25]. D3 dispersion corrections [26] to the functional were applied with Becke-Johnson damping [27]. The RIJ approximation to the Coulomb part [28] and COSX approximation [29] to the exchange part were used throughout all calculations with the def2-TZVP/J auxiliary basis set [30].

AC-QM/MM calculations were carried out at the one-body level, since exemplary two-body results for the main structure **1**, based on dataset **C**, provided essentially no changes in the geometry. A distance cutoff of 40 bohr was used to generate the cluster. NPA charges [31] were used for the electrostatic embedding, Lennard-Jones parameters from the Universal Force Field [32] (selenium only) and Charmm Generalised Force Field [33] (all other elements) for the further embedding.

Within the AC-QM/MM framework, the energy expression was modified to represent a disordered molecule in an ordered environment. The optimization is not carried out with respect to the cohesive energy E_{coh}, as defined in the AC-QM/MM model, but with respect to the embedded energy E_{emb}. The former is defined only for an unperturbed crystal, representing the stabilization of a molecule in the crystal compared to a gas phase molecule. It cannot be applied for this problem, as the underlying theory requires all molecules in the crystal to be the same. The embedded energy is apt to describe the relative stabilization of the disordered molecule in the frozen environment and allows for a reasonable optimization, but its absolute values do not have a physical meaning.

AC-QM/MM calculations were performed in two steps. Firstly, the crystal structure of **1**, based on dataset **C**, was optimized without restrictions, i.e. both the geometry and cell parameters were relaxed. The resulting structure was used as the foundation for further calculations. The environment was frozen: Geometries and embedding potentials were kept fixed at the values of this calculation.

For subsequent calculations, the QM molecule in the cluster was optimized in the frozen environment, starting from three different geometries and simulating six different electronic states. As starting geometry **1**, the experimental molecular structure was used unchanged. For **2**, the Se atoms were moved to the mean residual density peak positions of **A** to **F**. For **3** the Se-Se distance was elongated to 2.894 Å, the mean value of intra- and intermolecular Se-Se distance.

The electronic states taken into consideration were the singlet ground state (S_0) and first excited state (S_1), the lowest triplet state (T_0), and a broken symmetry state (BS). Furthermore, a singly charged cation and anion in their doublet ground states (D_0) were considered. The starting structure with Se atoms at the residual density peak positions was considered the best experimental estimation of the structure of **2**. In Table S14, the resulting geometries are compared to the experimental structure of **1** (Dataset **C**) and the starting conformation of **2** with Se at the residual density peak positions. Optimized structures of $(BnSe)_2$ **1**, BnSe-Se* *Bn **2** and BnSe* *SeBn **3** are labelled according to their electronic state, e.g. **1(S_0)**.

For the **1(S_0)** and **1(BS)** state, the starting geometry is retained. For **1(S_1)** and **1(T_0)** an elongation of the Se-Se bond is observed, resembling **3**. The cation **1(D_0^+)** leads to a significant torsion of the C-Se-Se-C dihedral angle, slightly smaller Se-Se and slightly larger C-Se distances do not resemble the experimental expectation. For the anion **1(D_0^-)**, an elongated Se-Se bond is observed, but quite similar residual geometry parameters.

Starting from the elongated Se-Se structure **3**, the optimizations **3(S_0)** to **3(D_0^-)** yield very similar, virtually unchanged structure for all electronic states with only negligible differences in geometry.

The optimization starting from the experimental residual density peak positions (**2**) provide interesting results. **2(S_0)** and **2(BS)** relax to the ground-state geometry like **1(S_0)**, as expected. For **2(S_1)** no energy minimum could be determined. A crossing with the S_0 energy surface was observed, thus indicating that at least in that structural region no energy minimum of the first excited singlet state is accessible. **2(T_0)** state did not relax to a **3** akin structure, as it had been the case for both other starting geometries. Although the starting structure was not retained, the general agreement of key geometry parameters between the experimental residual density peak positions and **2(T_0)** is quite reasonable. The Se-Se distance is notably longer (2.243 Å) compared to the residual density peak distance (2.170 Å) while one C-Se bond is shorter (2.004 Å) than in the experimental estimation (2.167 Å). However, the broken C-Se bond is very similar (3.132 Å) to the experimental estimation (3.155 Å) and one of the C-Se-Se angles is very close (100.8° to 93.3°). For the second angle (79.7°) the agreement to the experimental estimation (52.7°) is notably worse, as well for the C-Se-Se-C dihedral (109.2° to 126°). Nonetheless, it must be considered that in all parameters the results obtained from **2(T_0)** are not only in much better agreement than all other results, but also the deviations

from experimental **1** as well as **1(S_0)** are in the direction of the residual density peaks. Hence, it is not considered a clear evidence of the experimental structure representing a triplet state, but it strongly supports the assumption.

EPR parameters were calculated for the optimized triplet structures. Parameters for **2(T_0)** and **3(T_0)** were found to be notably different, thus allowing for an assignment of the experimental EPR results. The EPR spectra were calculated from the theoretical g tensors as described in the respective EPR part of this document.

Table S14.

Selected geometry parameters of optimized structures for different starting structures (Init) optimized in different electronic states (State) and compared to the experimental parameters (EXP), as well as the residual density peak positions as Se-atoms (EXPQ).

Init	State	*r*(Se-Se) [Å]	*r*(C-Se) [Å]		*a*(C-Se-Se) [°]		*d*(C-Se-Se-C) [°]
	EXP	2.315	1.984	1.984	101.1	101.1	93.6
	EXPQ	2.170	2.167	3.155	97.3	52.7	126.0
**1**	S_0	2.328	1.996	1.996	101.3	101.3	95.6
	S_1	2.785	1.986	1.985	92.9	93.0	112.2
	T_0	2.766	1.991	1.991	91.8	91.8	114.2
	BS	2.328	1.998	1.998	101.6	101.6	95.1
	D_0^+(Cation)	2.268	2.056	2.056	101.1	101.0	132.1
	D_0^-(Anion)	2.862	1.983	1.984	95.9	95.3	85.3
**2**	S_0	2.328	1.997	1.998	101.3	101.6	94.8
	S_1	-	-	-	-	-	-
	T_0	2.243	2.004	3.132	100.8	79.7	109.2
	BS	2.328	1.998	1.998	101.5	101.6	94.7
	D_0^+(Cation)	2.284	2.040	2.040	106.5	106.4	79.4
	D_0^-(Anion)	2.325	1.994	2.734	99.9	95.1	98.4
**3**	S_0	2.329	1.998	1.998	101.6	101.6	95.6
	S_1	2.819	1.979	1.977	100.2	102.2	73.4
	T_0	2.766	1.991	1.991	92.4	92.3	114.8
	BS	2.329	1.998	1.999	101.6	101.6	95.6
	D_0^+(Cation)	2.284	2.041	2.041	106.9	106.9	80.0
	D_0^-(Anion)	2.860	1.983	1.983	94.5	94.5	85.8

Table S15.

Local minimum structure obtained for the $\mathbf{S_0}$ state starting from structures **1**, **2** and **3**.

	X	Y	Z
Se	-0.08443767795831	0.53644073684133	1.16093614699555
C	1.33360022014297	-0.77820023415225	1.6574300981265
C	2.7216908825444	-0.25490223868549	1.54482846663533
C	3.56764531422931	-0.76017717288386	0.55940030228061
C	4.88257827065706	-0.31567067404859	0.46618562220482
C	5.36267688637808	0.63587006568352	1.35684692390982
C	4.51860685087095	1.15299793768733	2.34035770794664
C	3.20648960136741	0.71113792692357	2.43296632934162
H	1.08275882065016	-1.05192711238061	2.68185768498761
H	1.20046416105444	-1.64241657634464	1.01528993432772
H	3.20812390188472	-1.52626930601467	-0.11322406934569
H	5.52964059463643	-0.72666954952558	-0.29806441215899
H	6.38801582168072	0.97010582385005	1.30034060369019
H	4.89227392731387	1.89917091967061	3.03042969827996
H	2.55825052794264	1.0995389945189	3.20929837186513
Se	0.08467986418974	0.53668583084153	-1.1612839643761
C	-1.33352073175943	-0.77794274950994	-1.65768723212065
C	-2.72163845145832	-0.2547521624948	-1.54494684527975
C	-3.56749705597408	-0.76013131710601	-0.55948985764694
C	-4.88241838532578	-0.31563919987301	-0.46616499421712
C	-5.36262004524882	0.63595123870106	-1.35671282683651
C	-4.51867041582785	1.15309972768533	-2.34031939569954
C	-3.20655263685401	0.71126216368179	-2.43306386234767
H	-1.08284462077083	-1.05179157346494	-2.68211563122668
H	-1.19999747337496	-1.64195856390209	-1.01540120075325
H	-3.20795927597898	-1.52628893108829	0.11305867296479
H	-5.52943484777838	-0.72670393723925	0.29807346362195
H	-6.38796124105904	0.97013740473091	-1.30007141287455
H	-4.89244928409889	1.89922858758885	-3.0303805336086
H	-2.55842146055773	1.09965715098241	-3.2094865096417

Table S16.

Local minimum structure obtained for the **T_0** state starting from structures **1** and **3**.

	X	Y	Z
Se	-0.28703657649162	0.27842930671583	1.35301414433003
C	1.33329592141232	-0.80330082219269	1.76167074246717
C	2.67919655044501	-0.190840688961	1.55350788939592
C	3.51281537952833	-0.69321110133628	0.55126806875979
C	4.83366300218788	-0.26732977646751	0.45655712237815
C	5.33112715140616	0.66820749135534	1.35606298591754
C	4.49579796391482	1.1988840080103	2.33856107864185
C	3.17726956749904	0.77447964113851	2.43478013919094
H	1.20265246137747	-1.09382600351784	2.79970920092299
H	1.25317624368358	-1.68844209205659	1.13940378257068
H	3.14803153846709	-1.45790934786443	-0.12090558835231
H	5.4753995869772	-0.68710473715242	-0.3080205771142
H	6.36407299236387	0.9791709350971	1.30735200292094
H	4.88312691403336	1.93324119538435	3.03399468344285
H	2.54357581931719	1.15576062877315	3.22594714315132
Se	0.28717250245318	0.27707947589257	-1.35250217305662
C	-1.33375422117128	-0.80404928859104	-1.76143177448097
C	-2.67927000253562	-0.19102546885739	-1.55299800564082
C	-3.51299805965555	-0.69312363503572	-0.55067892471099
C	-4.83373094684319	-0.26721354973196	-0.45635488575703
C	-5.33114517213563	0.66805297268179	-1.35597997166609
C	-4.49558001190371	1.19883047082965	-2.33836975553417
C	-3.17700986517764	0.77438410753805	-2.43428489737888
H	-1.20313816400089	-1.09419371742365	-2.79956067720269
H	-1.25429622912158	-1.68970956139012	-1.13977736657148
H	-3.14813016247983	-1.45763317008019	0.12167410449574
H	-5.47562920411543	-0.68689264500948	0.30826408616547
H	-6.36411025250416	0.97891189025894	-1.30770516397876
H	-4.88268545724922	1.9331869164106	-3.03372826075672
H	-2.54315990746514	1.15563633850017	-3.22527862839218

Table S17.

Local minimum structure obtained for the $\mathbf{S_1}$ state starting from structures **1** and **3**.

	X	Y	Z
Se	-0.24965796055401	0.29751308040592	1.37024795671933
C	1.35021724751944	-0.80955471201583	1.7666432263559
C	2.69629484452528	-0.1945540700003	1.56710089851285
C	3.53913906623039	-0.68537687508936	0.56819374056834
C	4.85286849092381	-0.23803090556834	0.47334305285251
C	5.33617634615196	0.70425665017417	1.37256106665129
C	4.49158059189125	1.21996608969829	2.35465474838636
C	3.18034821375068	0.77515801306146	2.45032037575228
H	1.2167879214719	-1.10489148623091	2.80290270274459
H	1.26435798259395	-1.69170496620672	1.13977170057641
H	3.1832353423161	-1.45237935085731	-0.1061199831145
H	5.49790558392265	-0.64152894630358	-0.29713399877083
H	6.36167741839384	1.03608613346839	1.31897738594349
H	4.86600251521879	1.96488614849622	3.04628240360303
H	2.53781632864259	1.14881661473288	3.23802452670265
Se	0.25067809751762	0.29418228891468	-1.36919106666153
C	-1.35039341035239	-0.81067984004908	-1.76624184802258
C	-2.69620891513282	-0.19465933517719	-1.56674222193126
C	-3.53906363451362	-0.68533344085525	-0.56787982970642
C	-4.85294275263101	-0.23783341786718	-0.47334021308146
C	-5.33603173215618	0.7043197526182	-1.37252627242517
C	-4.49127078684661	1.22027651143734	-2.35494817000078
C	-3.17951267689388	0.77479783386716	-2.45005829471179
H	-1.2170719080953	-1.10592780195238	-2.80248166069971
H	-1.26553655114911	-1.69302259156404	-1.13944535798968
H	-3.18345710505335	-1.45222663070627	0.10661208738178
H	-5.49807262627909	-0.64116739169291	0.29709293713368
H	-6.36129406156407	1.036299699306	-1.31940829420577
H	-4.8652181907097	1.96475660467278	-3.04628234603925
H	-2.53725069597488	1.1484718375629	-3.23774433390788

Table S18.

Local minimum structure obtained for the **BS** state starting from structures **1**, **2** and **3**.

	X	Y	Z
Se	-0.0722821023757	0.54665520110934	1.16159407693578
C	1.3440246161222	-0.77495545365333	1.65103488151328
C	2.73473276302146	-0.25708453874873	1.54071487092453
C	3.58253123980471	-0.7587347213195	0.55323314097084
C	4.8972379130021	-0.31281228177798	0.46546676175373
C	5.37394934051616	0.63756551509859	1.35844284778532
C	4.52979477818841	1.15059238841106	2.34355344175008
C	3.21924859247915	0.70475577811851	2.4346552875477
H	1.08950556575296	-1.04798550558998	2.67491105877107
H	1.20841780166545	-1.63906981513882	1.00933271835502
H	3.22976596401116	-1.52235960327403	-0.12672419645668
H	5.54801765921259	-0.71885683017797	-0.29865886912645
H	6.39662091050289	0.97751628093856	1.30077437156193
H	4.90217829127073	1.89644619834533	3.03482705094321
H	2.57278210992515	1.09054185445618	3.21402233486683
Se	0.07263918009263	0.54690989173478	-1.16224561685872
C	-1.34331338634664	-0.77486684548755	-1.65162325313837
C	-2.73391591650851	-0.25692418041675	-1.5408271721398
C	-3.58135521836391	-0.75835782499237	-0.5534223960564
C	-4.89622519855969	-0.31280255782365	-0.46536577408524
C	-5.37303787078514	0.63758190786084	-1.35865460091696
C	-4.52909024812856	1.15067804399885	-2.34343307460508
C	-3.2183564659467	0.70519694560044	-2.43483267231416
H	-1.08911967981417	-1.04874281310055	-2.67533913102998
H	-1.2078465645484	-1.63867955465248	-1.00951528913917
H	-3.22838698695386	-1.52186785574778	0.12682043124134
H	-5.54682394495017	-0.71877600829291	0.29869783745037
H	-6.39588675094539	0.97724096730056	-1.30096850249132
H	-4.90138839232382	1.89667495980078	-3.03475150074455
H	-2.57190003108344	1.09116886517094	-3.21384586133156

Table S19.

Local minimum structure obtained for the $\mathbf{T_0}$ state starting from structure **2**.

	X	Y	Z
Se	0.04962073469256	0.38176898064962	1.03472448653789
C	1.44165689273631	-0.83961461507967	1.8015783814906
C	2.81397406713667	-0.30048863903776	1.64658220988033
C	3.63275719514322	-0.79639730844502	0.6323423777875
C	4.92903087509104	-0.31538506886489	0.48541185681565
C	5.41057036864892	0.66188124654725	1.34434028584918
C	4.59146281973371	1.17539616673592	2.34944306335281
C	3.29770534391446	0.69861374817174	2.49787604247372
H	1.14927767179354	-0.97231388569989	2.84045677534917
H	1.3390021340149	-1.77881076521387	1.26697001494657
H	3.27276682506973	-1.57727601652156	-0.02298293165116
H	5.56132110070166	-0.7146245714208	-0.29647262140759
H	6.42306708138291	1.01804894287839	1.24632067021442
H	4.97122581735057	1.9424873314562	3.01229509438902
H	2.66813258789777	1.08348300778465	3.29146476062447
Se	0.82059470906158	0.63856761454181	-1.05601062843474
C	-1.70794307645836	-1.12727450827582	-1.60266951612407
C	-2.91679533037689	-0.4516926842359	-1.46243415004105
C	-3.83949642359235	-0.84793204640426	-0.45820314395068
C	-5.09619595450471	-0.28767874712263	-0.40578672516595
C	-5.47702096755358	0.68342471836381	-1.33361325071959
C	-4.56338392473287	1.13558461608642	-2.2909645369469
C	-3.29821176382181	0.58869044102755	-2.35571603815664
H	-1.06180003532436	-0.94547373821841	-2.44685950084627
H	-1.4699493145959	-1.93899729139036	-0.93470205288033
H	-3.5625790277266	-1.63846913170714	0.22490272654203
H	-5.79863090281896	-0.62323622248519	0.34633541503512
H	-6.4789977536066	1.07095603386392	-1.32832143908196
H	-4.86254560532628	1.91026438631728	-2.98678562318544
H	-2.59904400734524	0.91130694211691	-3.11715113437846

Table S20.

Local minimum structure obtained for the $\mathbf{D_0^+}$ state starting from structures **1**, **2** and **3**.

D0(Cation)	X	Y	Z
Se	0.08151808430764	0.63660962129156	1.12854005493913
C	1.36523032492002	-0.87123430544662	1.61995143930582
C	2.72815813955057	-0.31776916088995	1.52583442958299
C	3.58240156283135	-0.76917988754165	0.51196389051378
C	4.88413707037266	-0.3033763872674	0.45133278595205
C	5.33657707449837	0.63720672362588	1.37957928184881
C	4.4820545094978	1.1166603751147	2.37223061354449
C	3.18463330577218	0.63970405981809	2.45327068419225
H	1.04937005243112	-1.13470402734799	2.62739007797291
H	1.20300941496167	-1.69026071151498	0.92970577292575
H	3.2477460888428	-1.52668838290663	-0.1831637593332
H	5.55363986927291	-0.68409631777627	-0.30766776942692
H	6.35714843187529	0.98733646633456	1.3427478760499
H	4.84117694864311	1.85205418127295	3.07973055923711
H	2.53341335324127	0.97826922657279	3.24987474475358
Se	-0.07523854953623	0.63835506908587	-1.15007853869065
C	-1.36339909210026	-0.86543654520871	-1.63897716898411
C	-2.72782691657575	-0.31594965254782	-1.53409149994958
C	-3.57606100935864	-0.77344671651972	-0.51791026055643
C	-4.87844312344137	-0.31017727744466	-0.44798262236283
C	-5.33636077638193	0.63640664684321	-1.36715345280369
C	-4.4906238175671	1.11615009969705	-2.3669674047235
C	-3.19250698367809	0.6423827935835	-2.45627570349804
H	-1.05346584751659	-1.1256727002093	-2.64914581250228
H	-1.19461177500677	-1.68632445810247	-0.95287504300705
H	-3.23665698297757	-1.53129827090864	0.17444307987973
H	-5.54378296152641	-0.69485606079413	0.31270807940147
H	-6.35184882319275	0.99788973211009	-1.31137964922935
H	-4.85538152745029	1.85111149632674	-3.07202557597198
H	-2.54656157208733	0.98677503997049	-3.25443845197536

Table S21.

Local minimum structure obtained for the $\mathbf{D_0^-}$ state starting from structures **1** and **3**.

	X	Y	Z
Se	0.02109925447738	0.83638501717823	1.43794651000453
C	1.35722298148691	-0.61535890984579	1.63894688444041
C	2.77605537895951	-0.17492858562385	1.52120276099272
C	3.59114566801847	-0.70276309001252	0.51977811827732
C	4.9281290918459	-0.32960424008016	0.4200443193813
C	5.46911091800454	0.58094192433632	1.3179690291304
C	4.65840994492766	1.12803650372461	2.31519970241297
C	3.32620697283247	0.75410755076314	2.41418940469211
H	1.18112798589935	-1.05854053118314	2.61827436936788
H	1.14631975188454	-1.35981176789538	0.8745526676091
H	3.1767471646235	-1.41777353017351	-0.17650327092116
H	5.54337090896488	-0.75480863948069	-0.36401247735342
H	6.50708972925877	0.87110271378152	1.24582938399875
H	5.0726275820607	1.85833769719146	3.00112800572294
H	2.69087573348315	1.18582273146424	3.17761191320986
Se	-0.00032507071486	0.82247653039884	-1.4236482478195
C	-1.34200891636323	-0.62896540031298	-1.59119824764069
C	-2.76182537179384	-0.18427171084453	-1.49156809757915
C	-3.59610682235856	-0.70787020335438	-0.50360573020349
C	-4.93293242235451	-0.32772239218486	-0.42578425979736
C	-5.45637316100684	0.58347765411678	-1.33388252953433
C	-4.62835376272048	1.1210577365541	-2.32203786072853
C	-3.29580703652469	0.74246485656358	-2.39648038369139
H	-1.16406908629085	-1.09526840825317	-2.55977928740212
H	-1.1349189328618	-1.3557867108848	-0.80868291831014
H	-3.19862976777456	-1.42748288647872	0.19778261379059
H	-5.56196394971829	-0.7495915601197	0.3496123980415
H	-6.49436417579866	0.8784949630258	-1.27876803728546
H	-5.02837567732076	1.84743008145966	-3.0211456385543
H	-2.6458508489081	1.16573755147508	-3.15178646285368

Table S22.

Local minimum structure obtained for the $\mathbf{D_0^-}$ state starting from structure **2**.

	X	Y	Z
Se	-0.00895610679142	0.61277194833974	1.2465326956252
C	1.37593627009406	-0.73219055432733	1.74629786394814
C	2.77008751496892	-0.23794034719643	1.60391068609881
C	3.57618277036368	-0.71617818506068	0.56965876352989
C	4.89614493444235	-0.29134221767254	0.44817913520401
C	5.42248237096484	0.62632872146134	1.34711411723552
C	4.61423895833233	1.13753307031676	2.36407871910347
C	3.30031893671444	0.70848172606713	2.48889868584238
H	1.15095053584113	-1.01087417320009	2.77428708560652
H	1.21916084083547	-1.58814718880902	1.09670566281983
H	3.17409100907353	-1.43511015413774	-0.12863845308654
H	5.51020781537419	-0.68381094393662	-0.35333700092642
H	6.4501297976265	0.94763908543139	1.26268638507154
H	5.01760609348459	1.87146148714312	3.05246236779899
H	2.67230433074091	1.1059186333366	3.27621840533428
Se	0.39255848141748	0.76973585802011	-1.03856906906916
C	-1.62459694561947	-0.93095458080556	-1.75575498227682
C	-2.87824944919074	-0.34584845437559	-1.55286018528757
C	-3.70274444518775	-0.75113873004979	-0.46686208077014
C	-4.99534114490302	-0.28894171090468	-0.34290620600351
C	-5.52100840942428	0.63158420060414	-1.25658716808777
C	-4.69817621455333	1.11408802757573	-2.28383151523264
C	-3.40636234168337	0.64944219399032	-2.43062269961346
H	-1.16170153776579	-0.90773170563126	-2.72676640414164
H	-1.3040859212863	-1.69884174294491	-1.06863465571182
H	-3.32179529282988	-1.48855600327332	0.22481906150593
H	-5.61555190617244	-0.6573987111747	0.46717352670837
H	-6.54533874433477	0.95430420178342	-1.17910344701576
H	-5.08733060530595	1.86195228657331	-2.96814975070119
H	-2.78134199297483	1.01697046171073	-3.23607323387426

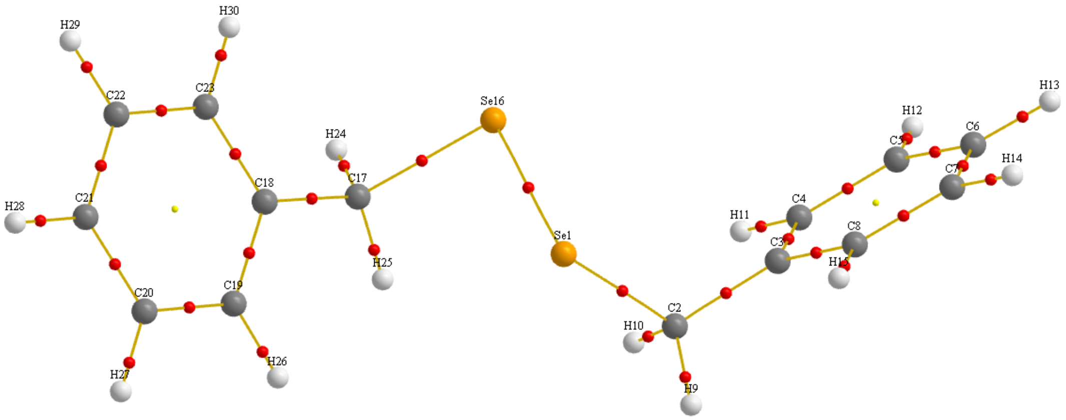

Fig. S31:

Bond Paths, BCP (red) and RCP (yellow) of **1(S$_0$)**.

Table S23.
BCP analysis of **1(S_0)**.

Atoms	Atom distance [Å]	Bond Path Length [Å]	$\rho(\mathbf{r}_{bcp})$ [e Å^{-3}]	$\Delta^2\rho(\mathbf{r}_{bcp})$ [e Å^{-5}]	$\varepsilon(\mathbf{r}_{bcp})$	Hessian Eigenvalues [a.u.]		
Se1 - Se16	2.32164	2.32195	0.725	-0.978	0.01	-0.0557	-0.0546	0.0889
Se1 - C2	1.99480	1.99506	0.930	-3.078	0.04	-0.0956	-0.0895	0.1175
C2 - C3	1.48865	1.48901	1.811	-17.453	0.02	-0.2881	-0.2775	0.1823
C3 - C4	1.39411	1.39415	2.156	-22.789	0.10	-0.3710	-0.3102	0.1808
C4 - C5	1.38980	1.38981	2.168	-23.151	0.10	-0.3740	-0.3148	0.1804
C5 - C6	1.38820	1.38822	2.172	-23.128	0.11	-0.3753	-0.3124	0.1798
C3 - C8	1.39853	1.39860	2.139	-22.521	0.10	-0.3669	-0.3086	0.1809
C6 - C7	1.39427	1.39428	2.147	-22.718	0.10	-0.3691	-0.3101	0.1803
C8 - H15	1.06979	1.06980	1.937	-24.716	0.01	-0.4172	-0.4113	0.2858
C7 - C8	1.38696	1.38696	2.176	-23.163	0.11	-0.3759	-0.3123	0.1795
C2 - H9	1.07636	1.07663	1.905	-23.732	0.00	-0.4063	-0.4028	0.2880
C2 - H10	1.07109	1.07129	1.932	-24.207	0.01	-0.4117	-0.4078	0.2880
C4 - H11	1.06678	1.06679	1.959	-25.397	0.01	-0.4290	-0.4226	0.2939
C5 - H12	1.06885	1.06885	1.934	-24.610	0.01	-0.4147	-0.4079	0.2821
C6 - H13	1.06508	1.06508	1.952	-25.018	0.01	-0.4212	-0.4137	0.2855
C7 - H14	1.06969	1.06969	1.931	-24.554	0.01	-0.4150	-0.4074	0.2832
Se16 - C17	1.99479	1.99506	0.930	-3.078	0.04	-0.0956	-0.0895	0.1175
C17 - C18	1.48871	1.48908	1.811	-17.448	0.02	-0.2880	-0.2774	0.1823
C18 - C19	1.39408	1.39411	2.156	-22.793	0.10	-0.3711	-0.3102	0.1808
C19 - C20	1.38991	1.38992	2.168	-23.141	0.10	-0.3739	-0.3147	0.1804
C20 - C21	1.38815	1.38816	2.172	-23.133	0.11	-0.3753	-0.3124	0.1798
C18 - C23	1.39853	1.39860	2.139	-22.520	0.10	-0.3669	-0.3086	0.1809
C21 - C22	1.39416	1.39417	2.147	-22.727	0.10	-0.3692	-0.3102	0.1803
C23 - H30	1.06974	1.06974	1.937	-24.722	0.01	-0.4173	-0.4114	0.2859
C22 - C23	1.38708	1.38709	2.175	-23.152	0.11	-0.3758	-0.3122	0.1795
C17 - H24	1.07635	1.07662	1.905	-23.732	0.00	-0.4063	-0.4028	0.2880
C17 - H25	1.07108	1.07128	1.932	-24.208	0.01	-0.4117	-0.4078	0.2880
C19 - H26	1.06676	1.06677	1.959	-25.399	0.01	-0.4290	-0.4226	0.2939
C20 - H27	1.06881	1.06881	1.934	-24.614	0.01	-0.4147	-0.4079	0.2821
C21 - H28	1.06517	1.06518	1.951	-25.010	0.01	-0.4211	-0.4135	0.2855
C22 - H29	1.06968	1.06968	1.931	-24.555	0.01	-0.4150	-0.4074	0.2832

Table S24.
Integrated Charges of **1(S$_0$).**

Name	q(A)	Name	q(A)	Name	q(A)	Name	q(A)
Se1	0.03			Se16	0.03		
C2	-0.14	H9	0.08	C17	-0.14	H24	0.08
C3	0.01	H10	0.04	C18	0.01	H25	0.04
C4	-0.04	H11	0.08	C19	-0.04	H26	0.08
C5	-0.05	H12	0.02	C20	-0.05	H27	0.02
C6	-0.05	H13	0.03	C21	-0.05	H28	0.03
C7	-0.05	H14	0.03	C22	-0.05	H29	0.03
C8	-0.03	H15	0.04	C23	-0.03	H30	0.04

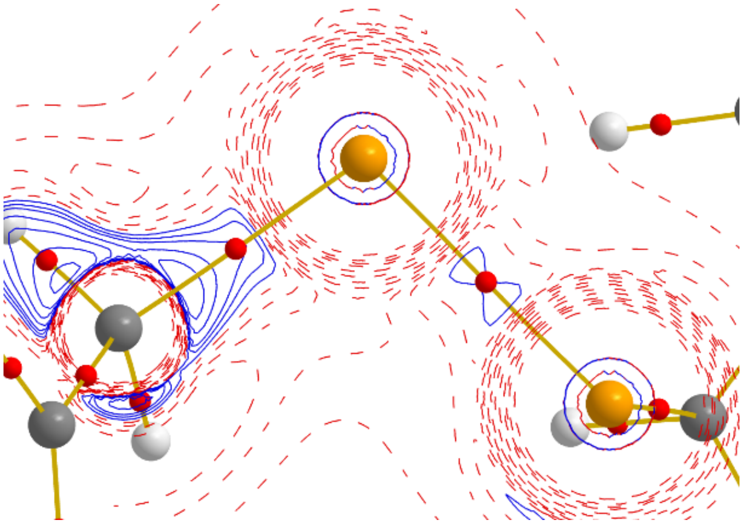

Fig. S32:
Laplacian map and BCPs (red) of **1(S$_0$)**. Isolevels +(red)/ −(blue) 0, 1, 2, 3, 5, 8, 13, 21, 34, 43, 55 and 89 eÅ^{-5}.

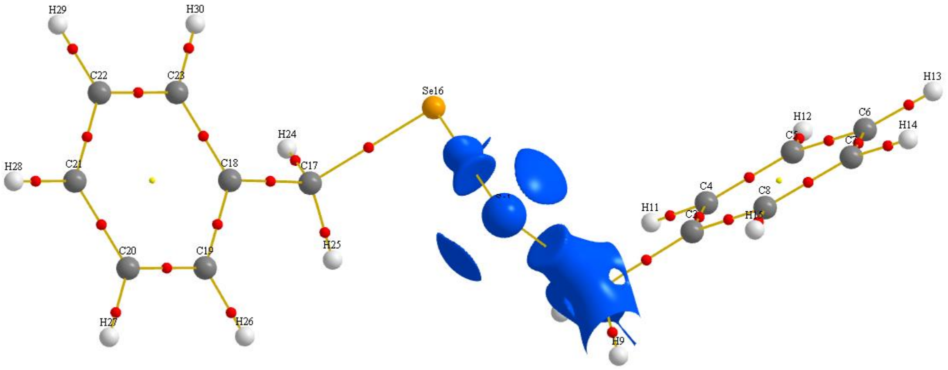

Fig. S33:
Se-VSCCs on the -0.01 a.u. laplacian isolevel of **1(S$_0$)**.

Residual Density Analysis Details

Model preparation

In order to achieve multipole populations unbiased by experimental errors, theoretical scattering factors were calculated based on the optimized structure **1(S_0)** using DenProp [34]. The atom positions were retained and only the multipole parameters were refined against the data, using the same restraints and local symmetry as for datasets **A** to **F**. The deformation density (Fig. S34) reproduces the electron densities features as found in Fig. S33. The residual density map level is reasonably low and mainly shows spherical features around the selenium positions (Fig. S34). This feature is common for the multipole-description of heavy atoms from calculated data and originates from the core-polarization [35].

Residual density determination

The determined multipole populations were applied to datasets **A** to **F**. Here, only atom positions, vibrational parameters and the scaling factor were refined against the experimental data, while the multipole populations were retained. The resulting residual density gives a good estimation of the differences between the theoretically expected and the experimentally determined density.

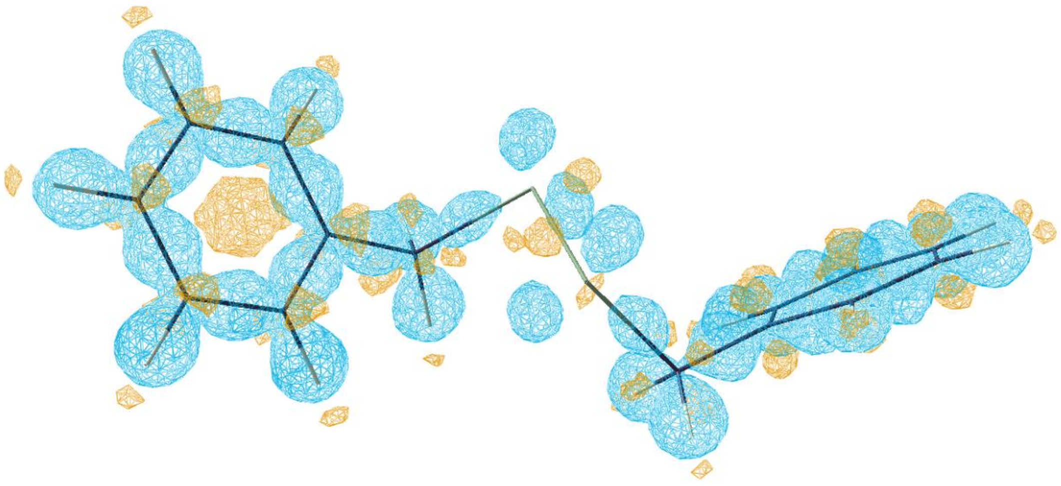

Fig. S34:

Deformation density map of the refined multipole model against calculated scattering factors of the optimized structure **1(S_0)**. Levels: +0.1 eÅ^{-1} (blue) and -0.1 eÅ^{-1} (orange).

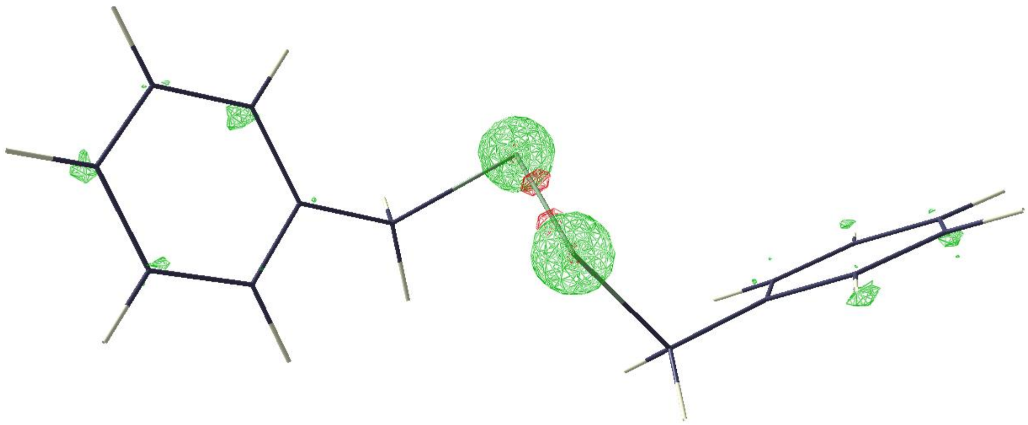

Fig. S35:

Residual density map of the refined multipole model against calculated scattering factors of the optimized structure **1(S_0)**. Levels: +0.1 eÅ^{-1} (green) and -0.1 eÅ^{-1} (red).

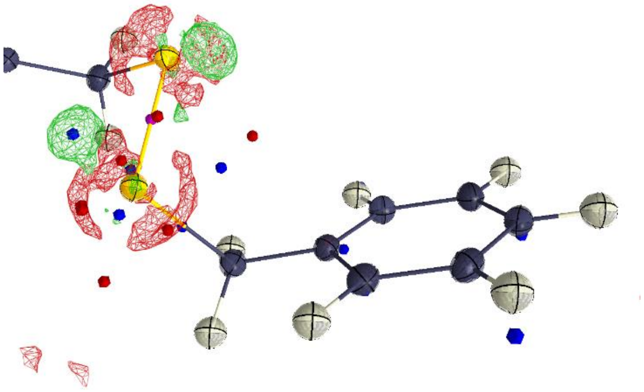

Fig. S36:

Residual density map of dataset **A** with theoretically determined aspherical scattering factors based on **1(S_0)**. Levels: +0.4 eÅ^{-1} (green) and -0.4 eÅ^{-1} (red).

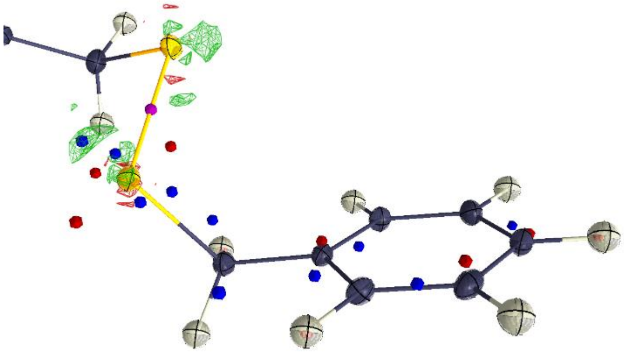

Fig. S37:

Residual density map of dataset **B** with theoretically determined aspherical scattering factors based on **1(S_0)**. Levels: +0.4 eÅ^{-1} (green) and -0.4 eÅ^{-1} (red).

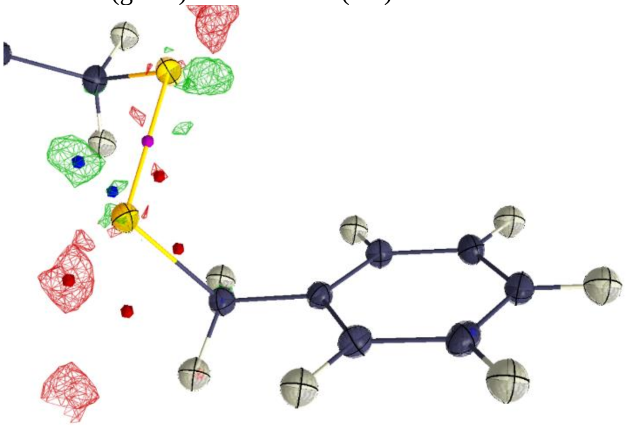

Fig. S38:

Residual density map of dataset **C** with theoretically determined aspherical scattering factors based on **1(S_0)**. Levels: +0.2 eÅ^{-1} (green) and -0.2 eÅ^{-1} (red).

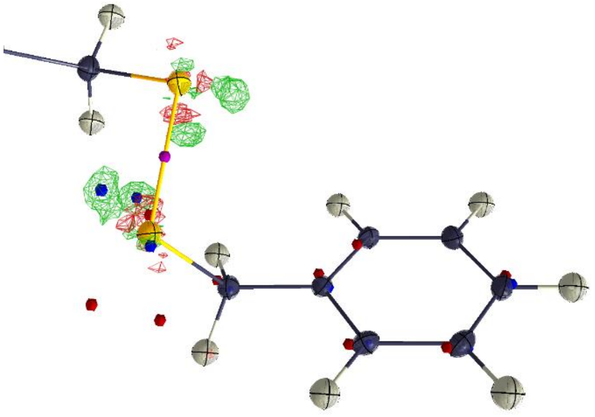

Fig. S39:

Residual density map of dataset **D** with theoretically determined aspherical scattering factors based on **1(S_0)**. Levels: +0.5 eÅ^{-1} (green) and -0.5 eÅ^{-1} (red).

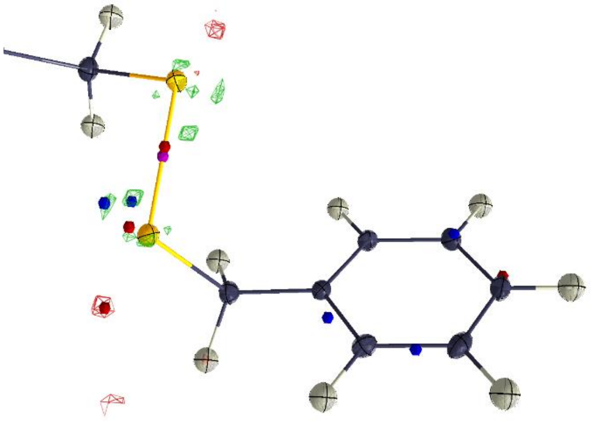

Fig. S40:

Residual density map of dataset **E** with theoretically determined aspherical scattering factors based on **1(S_0)**. Levels: +0.4 eÅ^{-1} (green) and -0.4 eÅ^{-1} (red).

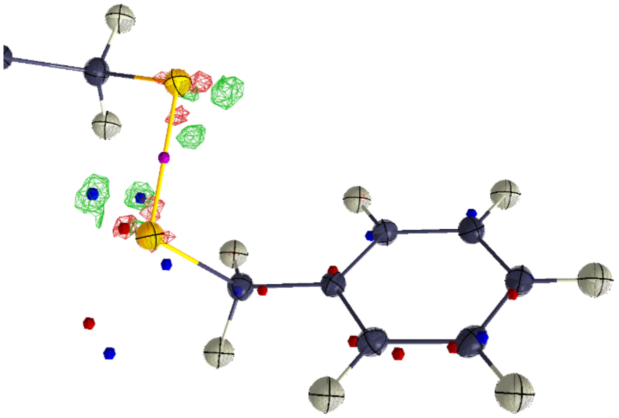

Fig. S41:

Residual density map of dataset **F** with theoretically determined aspherical scattering factors based on **1(S_0)**. Levels: +0.3 eÅ^{-1} (green) and -0.3 eÅ^{-1} (red).

Table S25.

Refinement results of dataset **A** to **F** with theoretical multipole populations based on **1(S_0)**.

Dataset	**A**	**B**	**C**	**D**	**E**	**F**
R1(F)	0.0265	0.0187	0.0181	0.0220	0.0166	0.0188
wR(F^2)	0.0309	0.0172	0.0200	0.0257	0.0203	0.0170
GOF(F)	4.9237	2.7309	2.0465	7.6497	3.3592	1.6780
$\Delta\rho_{max}$ [eÅ^{-3}]	1.717	0.721	0.541	1.409	0.436	0.587
$\Delta\rho_{min}$ [eÅ^{-3}]	-0.771	-0.826	-0.472	-1.070	-0.426	-0.594
E_{gross} [16]	30.0 e	28.7 e	29.5 e	30.8 e	31.7 e	19.9 e
Peak Pos. (Sphere size equals peak heights)	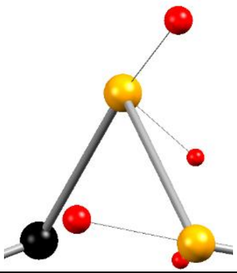	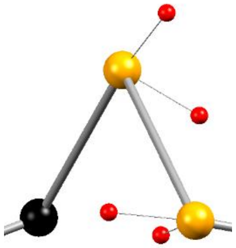	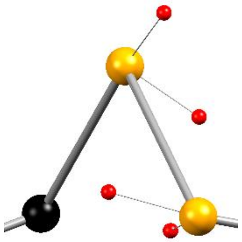	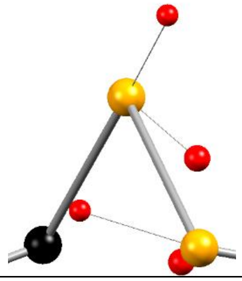	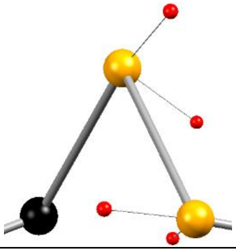	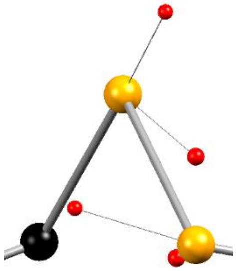
Peak heights 1 (in C-Se axis) [eÅ^{-3}]	1.72	0.72	0.54	0.99	0.44	0.55
Peak heights 2 (near Se-Se axis) [eÅ^{-3}]	0.72	0.61	0.49	1.41	0.38	0.59

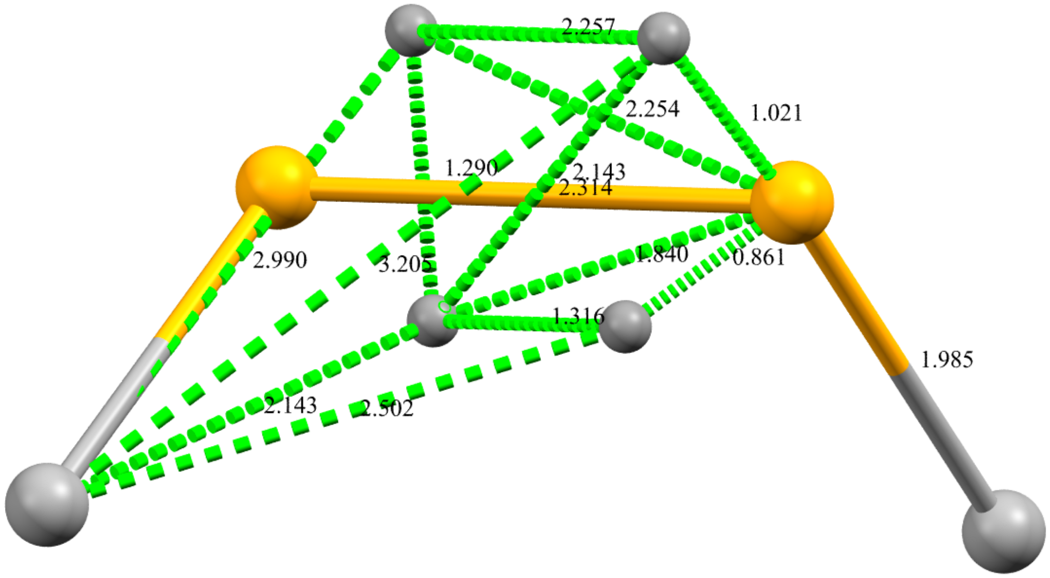

Fig. S42:
Mean residual density positions (grey) and distances (Å).

EPR Analysis Details

Clean samples of solid $(BnSe)_2$ were freshly recrystallized from tetrahydrofuran, ground thoroughly and were placed in Ø=5mm EPR tubes. UV- irradiated samples were irradiated for 6 hours with a 150 W Hg(Xe) arc lamp with a housing and power supply from LOT-Quantum Design GmbH. IR irradiation was cut off by a water filter. The sample was kept in liquid nitrogen. X-ray irradiated samples were irradiated in the full 'pink' beam of a 1200 W Rigaku MicroMax 007 rotating anode (Cu K_α), while cooled to approximately 100 K in a cold gaseous N_2 stream.

EPR spectra were collected within half an hour after irradiation, using a ELEXSYS CW-EPR spectrometer E500, equipped with a digital cryo-system ER 4131 VT at a microwave frequency of approximately 9.42 GHz, $1x10^{-4}$ G modulation amplitude, 100 kHz modulation frequency, and a microwave power of 5-6 mW in a temperature range between 142 and 290 K. The samples were re-evaluated after 1-2 days and in the case of the X-ray irradiated sample again after 30 days. The X-ray irradiated sample was re-evaluated after 60 days, using a Bruker E580 pulse EPR spectrometer operating at X band frequency for pulsed EPR measurements and a Bruker E500 ELEXYS CW-EPR spectrometer. Pulsed EPR measurements were recorded with a spin echo sequence using mw pulses of 20 and 40 ns for 90 and 180 degrees, respectively. The delays between first and second pulse were 420 ns (red spectrum) and 3080 ns (blue spectrum) to separate species with differing spin-spin-relaxation times. Spectra were recorded at 298 K at a microwave frequency of 9.60 GHz, acquiring two scans with 500 shot/point. CW EPR spectra were recorded at a mw frequency of 9.84 GHz, 0.2 G modulation amplitude, 100 kHz modulation frequency, and a mw power of 6-7 mW.

Fig. S43B shows the EPR signal of a UV-irradiated sample, which was not fully submerged in liquid nitrogen during the irradiation. The red discoloration of the sample in that area and the otherwise not detectable signal at g = 2.9 brought us to the conclusion to amorphous selenium [36] in contrast to other contemporary studies [37].

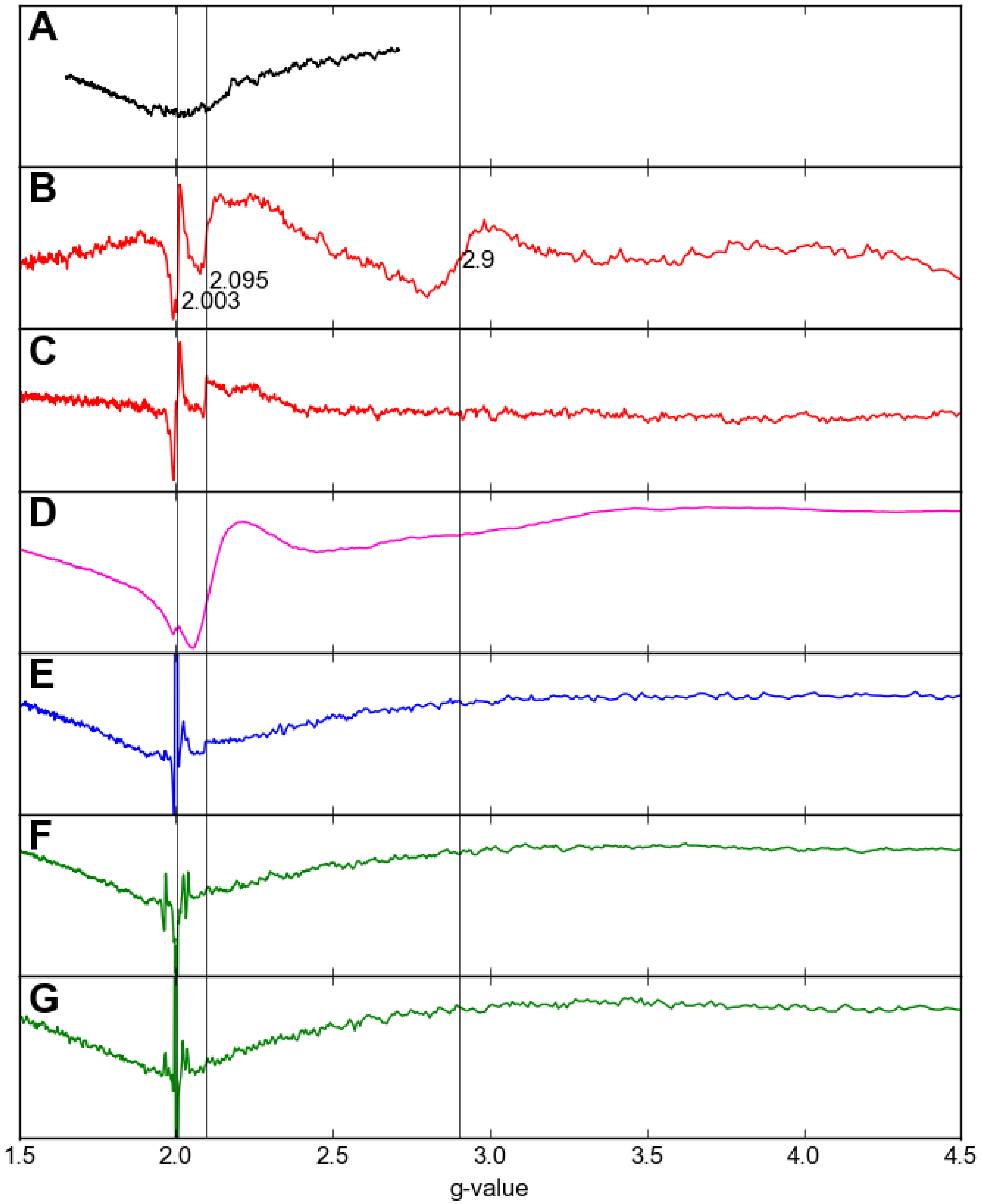

Fig. S43:

EPR signals at low resolution of an un-irradiated sample (**A**), a UV irradiated sample, not fully submerged in liquid nitrogen (**B**), a UV-irradiated sample (**C**) the same sample after 72h at RT (**D**), an X-ray irradiated sample (**E**), the same sample after 24 h (**F**) and 30 d (**G**) at RT.

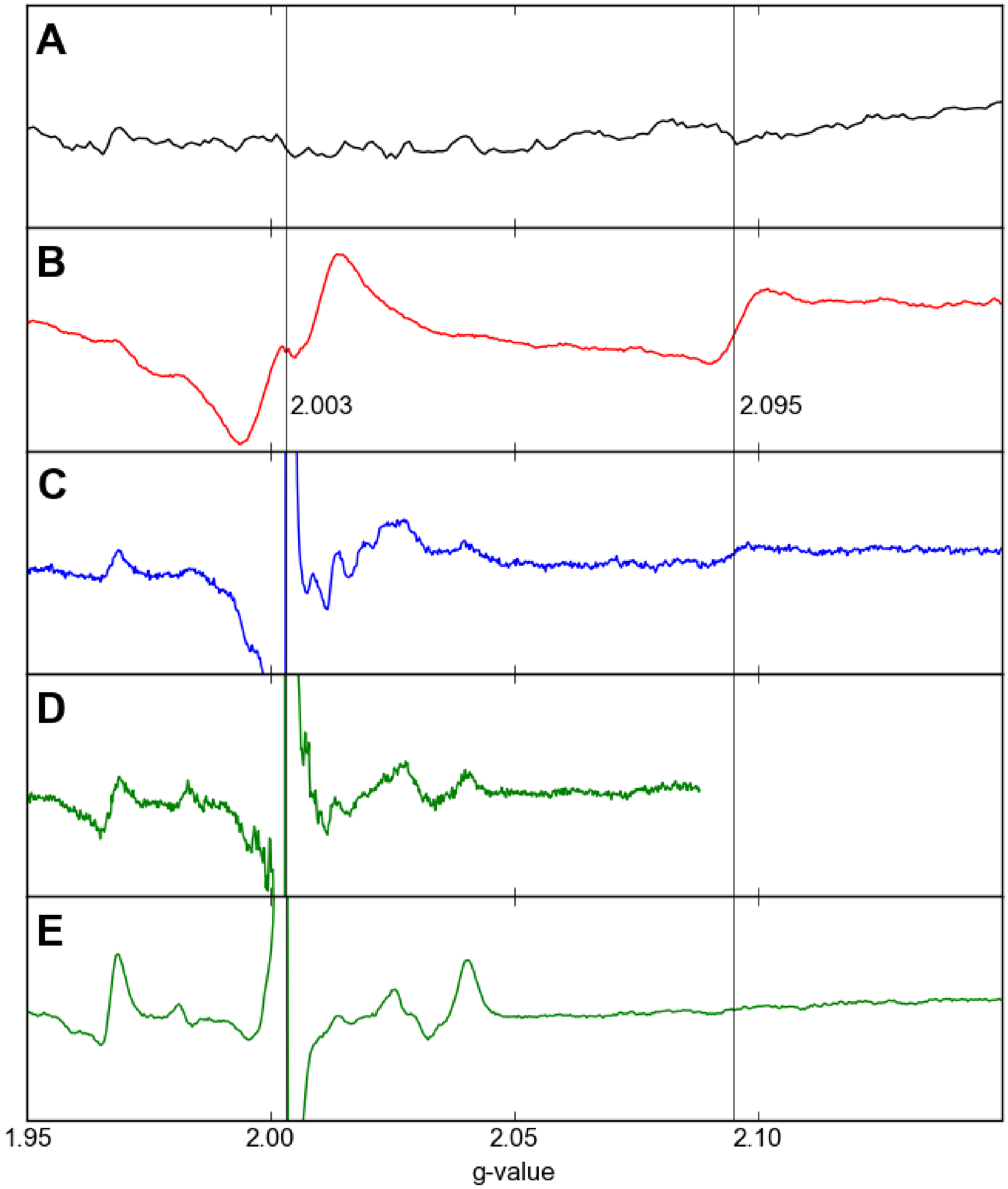

Fig. S44:

EPR signals at medium resolution of an un-irradiated sample (**A**), a UV-irradiated sample (**B**) an X-ray irradiated sample (**C**), the same sample after 24 h (**D**) and 30 d (**E**) at RT.

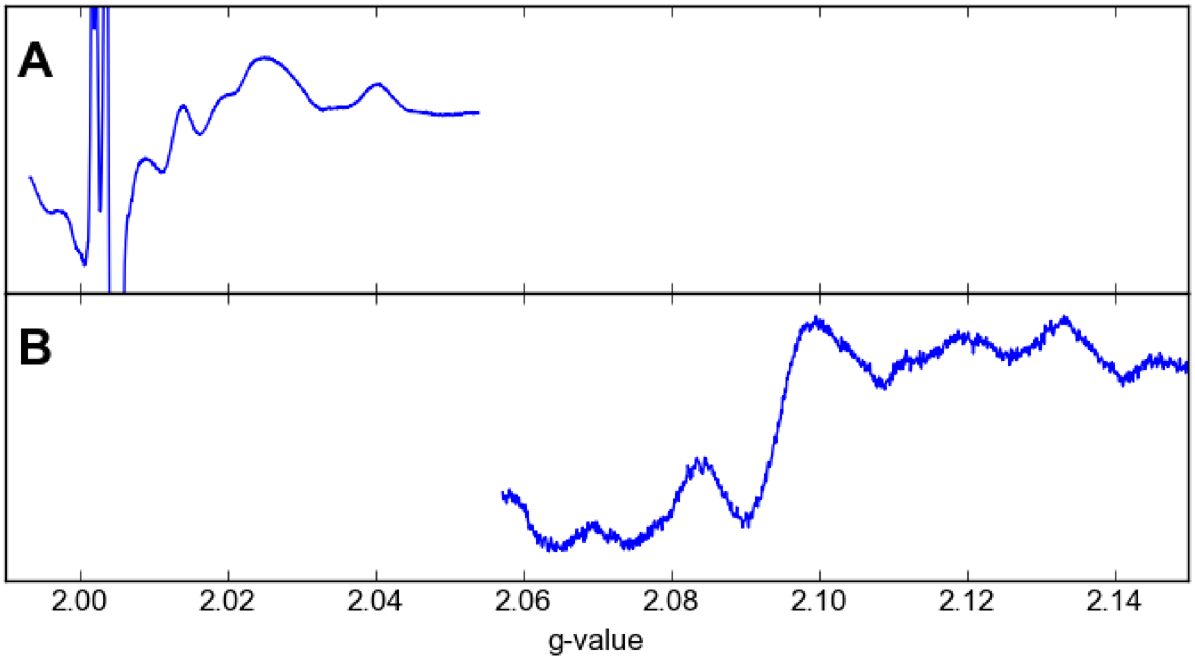

Fig. S45:
EPR signals at high resolution of an X-ray irradiated sample (**A, B**).

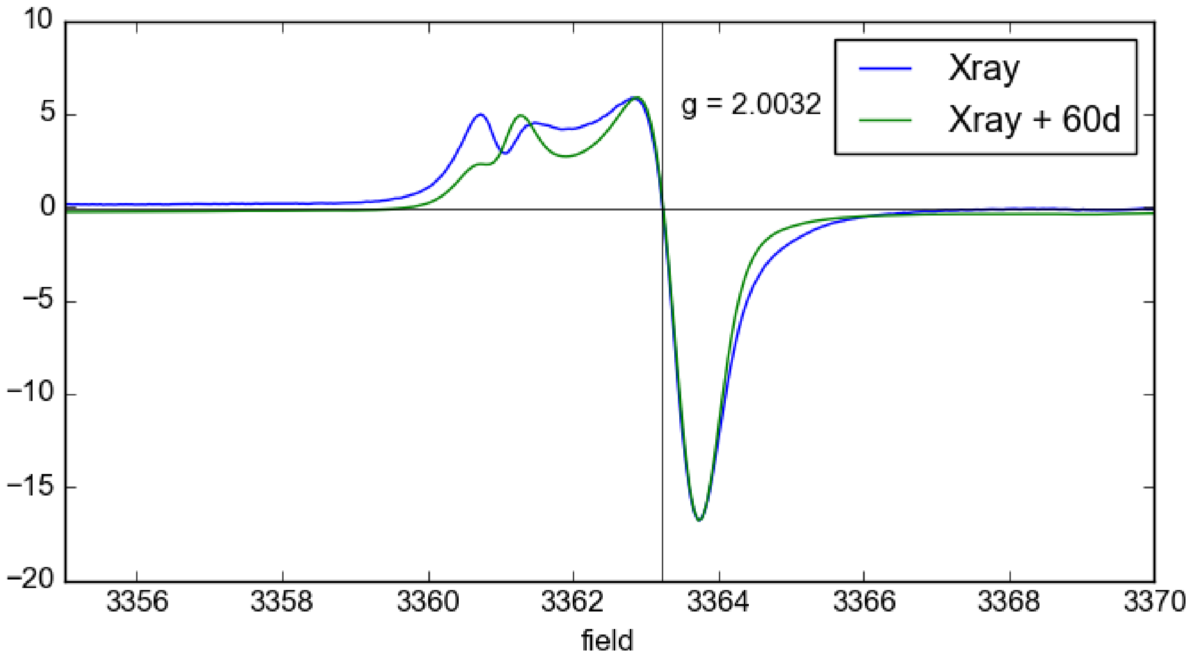

Fig. S46:
Detailed EPR signals of the main peaks of an X-ray irradiated sample and the same sample after 60 days.

[1] Guido van Rossum, *Python*, Python Software Foundation.
[2] E. B. Knudsen, H. O. Sørensen, J. P. Wright, G. Goret, J. Kieffer, *J. Appl. Cryst.* **2013**, *46*, 537.
[3] Bruker AXS Inc., *SAINT*, Madison, **2016**.
[4] L. Krause, R. Herbst-Irmer, G. M. Sheldrick, D. Stalke, *J. Appl. Cryst.* **2015**, *48*, 3.
[5] G. M. Sheldrick, *XPREP*, Göttingen, **2015**.
[6] G. M. Sheldrick, *Acta Crystallogr.* **2015**, *A71*, 3.
[7] G. M. Sheldrick, *Acta Crystallogr.* **2015**, *C71*, 3.
[8] C. B. Hübschle, G. M. Sheldrick, B. Dittrich, *J. Appl. Cryst.* **2011**, *44*, 1281.
[9] A. Volkov, P. Macchi, L. J. Farrugia, C. Gatti, P. R. Mallinson, T. Richter, T. Koritsanszky, *XD2006. A Computer Program Package for Multipole Refinement, Topological Analysis of Charge Densities and Evaluation of Intermolecular Energies from Experimental and Theoretical Structure Factors*, **2006**.
[10] a) B. Zarychta, V. Pichon-Pesme, B. Guillot, C. Lecomte, C. Jelsch, *Acta Crystallogr. Sec. A* **2007**, *63*, 108; b) C. Jelsch, B. Guillot, A. Lagoutte, C. Lecomte, *J. Appl. Cryst.* **2001**, *38*, 38; c) B. Guillot, L. Viry, R. Guillot, C. Lecomte, C. Jelsch, *J. Appl. Cryst.* **2001**, *34*, 214; d) C. Jelsch, B. Guillot, F. Pascale, A. Lagoutte, B. Fournier, S. Domagala, S. Alexandre, C. Iordache, *MoPro1805*, Lorraine, **2018**.
[11] L. Krause, B. Niepötter, C. J. Schürmann, D. Stalke, R. Herbst-Irmer, *IUCrJ* **2017**, *4*, 420.
[12] W. F. Kuhs, *Acta Crystallogr. Sec. A* **1992**, *48*, 80.
[13] Adam Stash, *DRKplot*, Moscow, **2007**.
[14] V. V. Zhurov, E. A. Zhurova, A. A. Pinkerton, *J. Appl. Cryst.* **2008**, *41*, 340.
[15] V. Zavodnik, A. Stash, V. Tsirelson, R. de Vries, D. Feil, *Acta Crystallogr. Sec. B* **1999**, *55*, 45.
[16] K. Meindl, J. Henn, *Acta Crystallogr. Sec. A* **2008**, *64*, 404.
[17] M. J. Turner, J. J. McKinnon, S. K. Wolff, D. J. Grimwood, P. R. Spackman, D. Jayatilaka, M. A. Spackman, *CrystalExplorer 17.5*, University of Western Australia, **2018**.
[18] C. B. Hübschle, B. Dittrich, *J. Appl. Cryst.* **2011**, *44*, 238.
[19] R. Herbst-Irmer, J. Henn, J. J. Holstein, C. B. Hübschle, B. Dittrich, D. Stern, D. Kratzert, D. Stalke, *J. Phys. Chem. A* **2013**, *117*, 633.
[20] S. C. Abrahams, E. T. Keve, *Acta Crystallogr. Sec. A* **1971**, *27*, 157.
[21] T. Teuteberg, M. Eckhoff, R. A. Mata, *A Full Additive QM/MM Scheme for the Computation of Molecular Crystals with Extension to Many-Body Expansions*, **2018**.
[22] a) *ChemShell. a Computational Chemistry Shell*; b) S. Metz, J. Kästner, A. A. Sokol, T. W. Keal, P. Sherwood, *Wiley Interdisciplinary Reviews: Computational Molecular Science* **2014**, *4*, 101; c) P. Sherwood, A. H. de Vries, M. F. Guest, G. Schreckenbach, C.R. A. Catlow, S. A. French, A. A. Sokol, S. T. Bromley, W. Thiel, A. J. Turner et al., *Journal of Molecular Structure: THEOCHEM* **2003**, *632*, 1.
[23] a) F. Neese, *ORCA. An ab initio, DFT and semiempirical SCF-MO package*; b) P. Y. Ayala, K. N. Kudin, G. E. Scuseria, *J. Chem. Phys.* **2001**, *115*, 9698.
[24] A. D. Becke, *J. Chem. Phys.* **1993**, *98*, 5648.
[25] F. Weigend, R. Ahlrichs, *Phys Chem Chem Phys* **2005**, *7*, 3297.
[26] S. Grimme, J. Antony, S. Ehrlich, H. Krieg, *J. Chem. Phys.* **2010**, *132*, 154104.
[27] a) A. D. Becke, E. R. Johnson, *J. Chem. Phys.* **2005**, *123*, 154101; b) E. R. Johnson, A. D. Becke, *J. Chem. Phys.* **2005**, *123*, 24101; c) E. R. Johnson, A. D. Becke, *J. Chem. Phys.* **2006**, *124*, 174104.
[28] a) E. J. Baerends, D. E. Ellis, P. Ros, *Chem Phys* **1973**, *2*, 41; b) J. L. Whitten, *J. Chem. Phys.* **1973**, *58*, 4496.
[29] F. Neese, F. Wennmohs, A. Hansen, U. Becker, *Chem. Phys.* **2009**, *356*, 98.
[30] K. Eichkorn, O. Treutler, H. Öhm, M. Häser, R. Ahlrichs, *Chem. Phys. Lett.* **1995**, *240*, 283.
[31] A. E. Reed, R. B. Weinstock, F. Weinhold, *J. Chem. Phys.* **1985**, *83*, 735.
[32] A. K. Rappe, C. J. Casewit, K. S. Colwell, W. A. Goddard, W. M. Skiff, *J. Am. Chem. Soc.* **1992**, *114*, 10024.
[33] K. Vanommeslaeghe, E. Hatcher, C. Acharya, S. Kundu, S. Zhong, J. Shim, E. Darian, O. Guvench, P. Lopes, I. Vorobyov et al., *J. Comput. Chem.* **2010**, *31*, 671.
[34] A. Volkov, H. F. King, T. Koritsanszky, *DenProp version 04/01/16*, **2016**.
[35] A. Fischer, D. Tiana, W. Scherer, K. Batke, G. Eickerling, H. Svendsen, N. Bindzus, B. B. Iversen, *J. Phys. Chem. A* **2011**, *115*, 13061.
[36] P. I. Sampath, *J. Chem. Phys.* **1966**, *45*, 3519.
[37] S. Nehzati, N. V. Dolgova, D. Sokaras, T. Kroll, J. J. H. Cotelesage, I. J. Pickering, G. N. George, *Inorg. Chem.* **2018**, *57*, 10867.
