# Supplementary material for: Trapping X‐ray Radiation Damage from Homolytic Se−C Bond Cleavage in BnSeSeBn Crystals (Bn=benzyl, CH2C6H5)
Source: Angew Chem Int Ed Engl. 2022 May 3;61(26):e202203665. doi: 10.1002/anie.202203665 (PMC9320817; doi:10.1002/anie.202203665)

checkCIF/PLATON report

Structure factors have been supplied for datablock(s) A_shelx

THIS REPORT IS FOR GUIDANCE ONLY. IF USED AS PART OF A REVIEW PROCEDURE FOR PUBLICATION, IT SHOULD NOT REPLACE THE EXPERTISE OF AN EXPERIENCED CRYSTALLOGRAPHIC REFEREE.

No syntax errors found. CIF dictionary Interpreting this report

Datablock: A_shelx

Bond precision:	C-C = 0.0008 A		Wavelength=0.71073
Cell:	a=13.7033(10)	b=8.0012(6)	c=11.4769(8)
	alpha=90	beta=99.293(2)	gamma=90
Temperature:	100 K		

	Calculated	Reported
Volume	1241.85(16)	1241.84(16)
Space group	C 2/c	C 2/c
Hall group	-C 2yc	-C 2yc
Moiety formula	C14 H14 Se2	C14 H14 Se2
Sum formula	C14 H14 Se2	C14 H14 Se2
Mr	340.17	340.17
Dx,g cm-3	1.819	1.819
Z	4	4
Mu (mm-1)	5.923	5.923
F000	664.0	664.0
F000'	663.54	
h,k,lmax	30,17,25	30,17,25
Nref	7173	7162
Tmin,Tmax	0.173,0.447	0.251,0.454
Tmin'	0.076	

Correction method= # Reported T Limits: Tmin=0.251 Tmax=0.454
AbsCorr = MULTI-SCAN

Data completeness= 0.998 Theta(max)= 52.248

R(reflections)= 0.0225(5863) wR2(reflections)= 0.0716(7162)

S = 1.075 Npar= 73

The following ALERTS were generated. Each ALERT has the format
test-name_ALERT_alert-type_alert-level.
Click on the hyperlinks for more details of the test.

Alert level B

```
PLAT230_ALERT_2_B Hirshfeld Test Diff for    Se1      --C1       .       10.1 s.u.
```

Author Response: This results from the radiation damage.

Alert level C

```
PLAT906_ALERT_3_C Large K Value in the Analysis of Variance ......      2.816 Check
PLAT911_ALERT_3_C Missing FCF Refl Between Thmin & STh/L=    0.600          2 Report
PLAT971_ALERT_2_C Check Calcd Resid. Dens.  1.12Ang From Se1              1.78 eA-3
```

Alert level G

```
PLAT883_ALERT_1_G No Info/Value for _atom_sites_solution_primary .     Please Do !
PLAT912_ALERT_4_G Missing # of FCF Reflections Above STh/L=  0.600         10 Note
PLAT933_ALERT_2_G Number of HKL-OMIT Records in Embedded .res File          3 Note
PLAT978_ALERT_2_G Number C-C Bonds with Positive Residual Density.          7 Info
```

0 **ALERT level A** = Most likely a serious problem - resolve or explain
1 **ALERT level B** = A potentially serious problem, consider carefully
3 **ALERT level C** = Check. Ensure it is not caused by an omission or oversight
4 **ALERT level G** = General information/check it is not something unexpected

1 ALERT type 1 CIF construction/syntax error, inconsistent or missing data
4 ALERT type 2 Indicator that the structure model may be wrong or deficient
2 ALERT type 3 Indicator that the structure quality may be low
1 ALERT type 4 Improvement, methodology, query or suggestion
0 ALERT type 5 Informative message, check

It is advisable to attempt to resolve as many as possible of the alerts in all categories. Often the minor alerts point to easily fixed oversights, errors and omissions in your CIF or refinement strategy, so attention to these fine details can be worthwhile. In order to resolve some of the more serious problems it may be necessary to carry out additional measurements or structure refinements. However, the purpose of your study may justify the reported deviations and the more serious of these should normally be commented upon in the discussion or experimental section of a paper or in the "special_details" fields of the CIF. checkCIF was carefully designed to identify outliers and unusual parameters, but every test has its limitations and alerts that are not important in a particular case may appear. Conversely, the absence of alerts does not guarantee there are no aspects of the results needing attention. It is up to the individual to critically assess their own results and, if necessary, seek expert advice.

Publication of your CIF in IUCr journals

A basic structural check has been run on your CIF. These basic checks will be run on all CIFs submitted for publication in IUCr journals (*Acta Crystallographica*, *Journal of Applied Crystallography*, *Journal of Synchrotron Radiation*); however, if you intend to submit to *Acta Crystallographica Section C* or *E* or *IUCrData*, you should make sure that full publication checks are run on the final version of your CIF prior to submission.

Publication of your CIF in other journals

Please refer to the *Notes for Authors* of the relevant journal for any special instructions relating to CIF submission.

PLATON version of 19/02/2022; check.def file version of 19/02/2022

Datablock A_shelx - ellipsoid plot

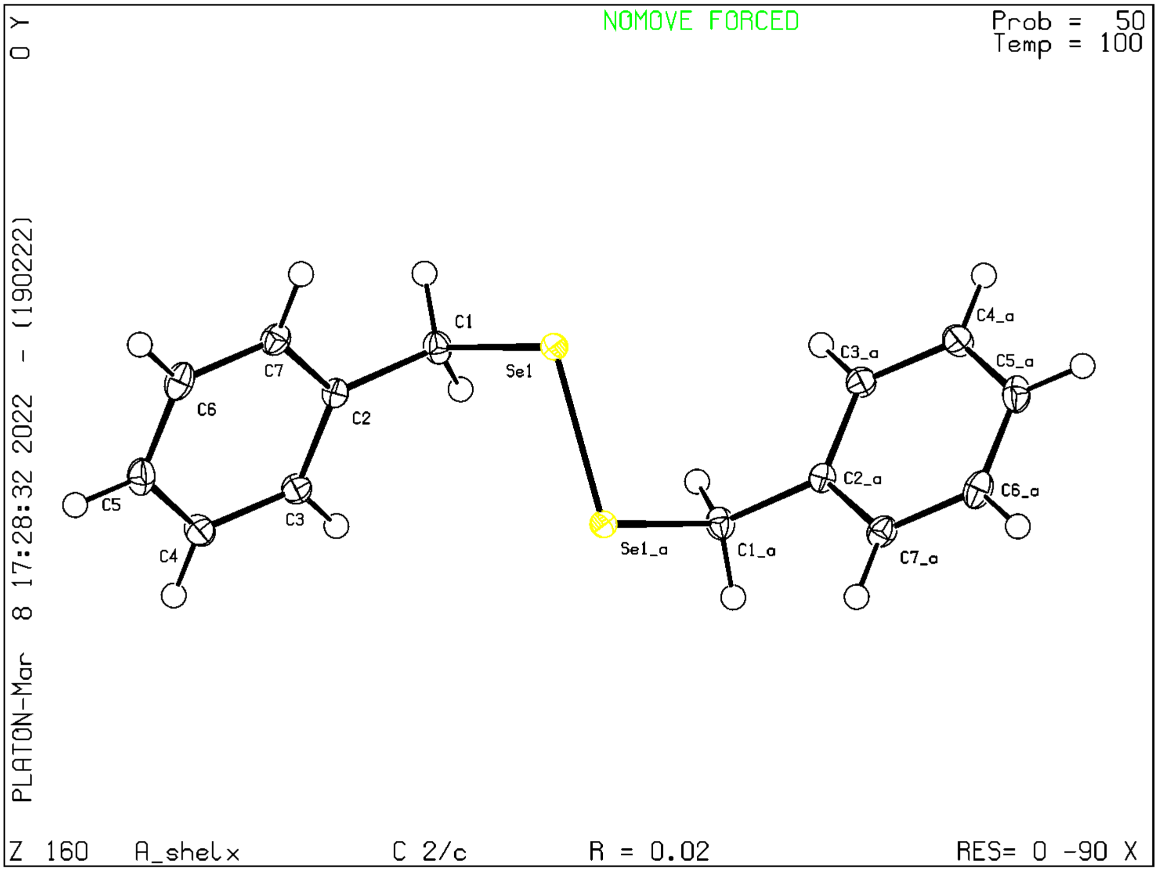

checkCIF/PLATON report

Structure factors have been supplied for datablock(s) B_shelx

THIS REPORT IS FOR GUIDANCE ONLY. IF USED AS PART OF A REVIEW PROCEDURE FOR PUBLICATION, IT SHOULD NOT REPLACE THE EXPERTISE OF AN EXPERIENCED CRYSTALLOGRAPHIC REFEREE.

No syntax errors found.   CIF dictionary   Interpreting this report

Datablock: B_shelx

Bond precision:	C-C = 0.0007 A		Wavelength=0.56086
Cell:	a=13.717(2)	b=8.0127(13)	c=11.4944(18)
	alpha=90	beta=99.303(7)	gamma=90
Temperature:	100 K		

	Calculated	Reported
Volume	1246.7(3)	1246.8(3)
Space group	C 2/c	C 2/c
Hall group	-C 2yc	-C 2yc
Moiety formula	C14 H14 Se2	C14 H14 Se2
Sum formula	C14 H14 Se2	C14 H14 Se2
Mr	340.17	340.17
Dx,g cm-3	1.812	1.812
Z	4	4
Mu (mm-1)	3.129	3.129
F000	664.0	664.0
F000'	665.94	
h,k,lmax	30,17,25	30,17,25
Nref	7201	7196
Tmin,Tmax	0.384,0.573	0.315,0.440
Tmin'	0.356	

Correction method= # Reported T Limits: Tmin=0.315 Tmax=0.440
AbsCorr = MULTI-SCAN

Data completeness= 0.999   Theta(max)= 38.612

R(reflections)= 0.0185(5391)   wR2(reflections)= 0.0644(7196)

S = 1.065   Npar= 73

The following ALERTS were generated. Each ALERT has the format
test-name_ALERT_alert-type_alert-level.
Click on the hyperlinks for more details of the test.

Alert level B

```
PLAT230_ALERT_2_B Hirshfeld Test Diff for    Se1      --C1       .        8.5 s.u.
```

Author Response: This results from the radiation damage.

Alert level C

```
PLAT094_ALERT_2_C Ratio of Maximum / Minimum Residual Density ....       2.03 Report
PLAT905_ALERT_3_C Negative K value in the Analysis of Variance ...     -0.001 Report
PLAT911_ALERT_3_C Missing FCF Refl Between Thmin & STh/L=    0.600          4 Report
```

Alert level G

```
PLAT019_ALERT_1_G _diffrn_measured_fraction_theta_full/*_max < 1.0      0.997 Report
PLAT883_ALERT_1_G No Info/Value for _atom_sites_solution_primary .     Please Do !
PLAT912_ALERT_4_G Missing # of FCF Reflections Above STh/L=  0.600          2 Note
PLAT933_ALERT_2_G Number of HKL-OMIT Records in Embedded .res File          4 Note
PLAT978_ALERT_2_G Number C-C Bonds with Positive Residual Density.          7 Info
```

0 **ALERT level A** = Most likely a serious problem - resolve or explain
1 **ALERT level B** = A potentially serious problem, consider carefully
3 **ALERT level C** = Check. Ensure it is not caused by an omission or oversight
5 **ALERT level G** = General information/check it is not something unexpected

2 ALERT type 1 CIF construction/syntax error, inconsistent or missing data
4 ALERT type 2 Indicator that the structure model may be wrong or deficient
2 ALERT type 3 Indicator that the structure quality may be low
1 ALERT type 4 Improvement, methodology, query or suggestion
0 ALERT type 5 Informative message, check

It is advisable to attempt to resolve as many as possible of the alerts in all categories. Often the minor alerts point to easily fixed oversights, errors and omissions in your CIF or refinement strategy, so attention to these fine details can be worthwhile. In order to resolve some of the more serious problems it may be necessary to carry out additional measurements or structure refinements. However, the purpose of your study may justify the reported deviations and the more serious of these should normally be commented upon in the discussion or experimental section of a paper or in the "special_details" fields of the CIF. checkCIF was carefully designed to identify outliers and unusual parameters, but every test has its limitations and alerts that are not important in a particular case may appear. Conversely, the absence of alerts does not guarantee there are no aspects of the results needing attention. It is up to the individual to critically assess their own results and, if necessary, seek expert advice.

Publication of your CIF in IUCr journals

A basic structural check has been run on your CIF. These basic checks will be run on all CIFs submitted for publication in IUCr journals (*Acta Crystallographica*, *Journal of Applied Crystallography*, *Journal of Synchrotron Radiation*); however, if you intend to submit to *Acta Crystallographica Section C* or *E* or *IUCrData*, you should make sure that full publication checks are run on the final version of your CIF prior to submission.

Publication of your CIF in other journals

Please refer to the *Notes for Authors* of the relevant journal for any special instructions relating to CIF submission.

PLATON version of 19/02/2022; check.def file version of 19/02/2022

Datablock B_shelx - ellipsoid plot

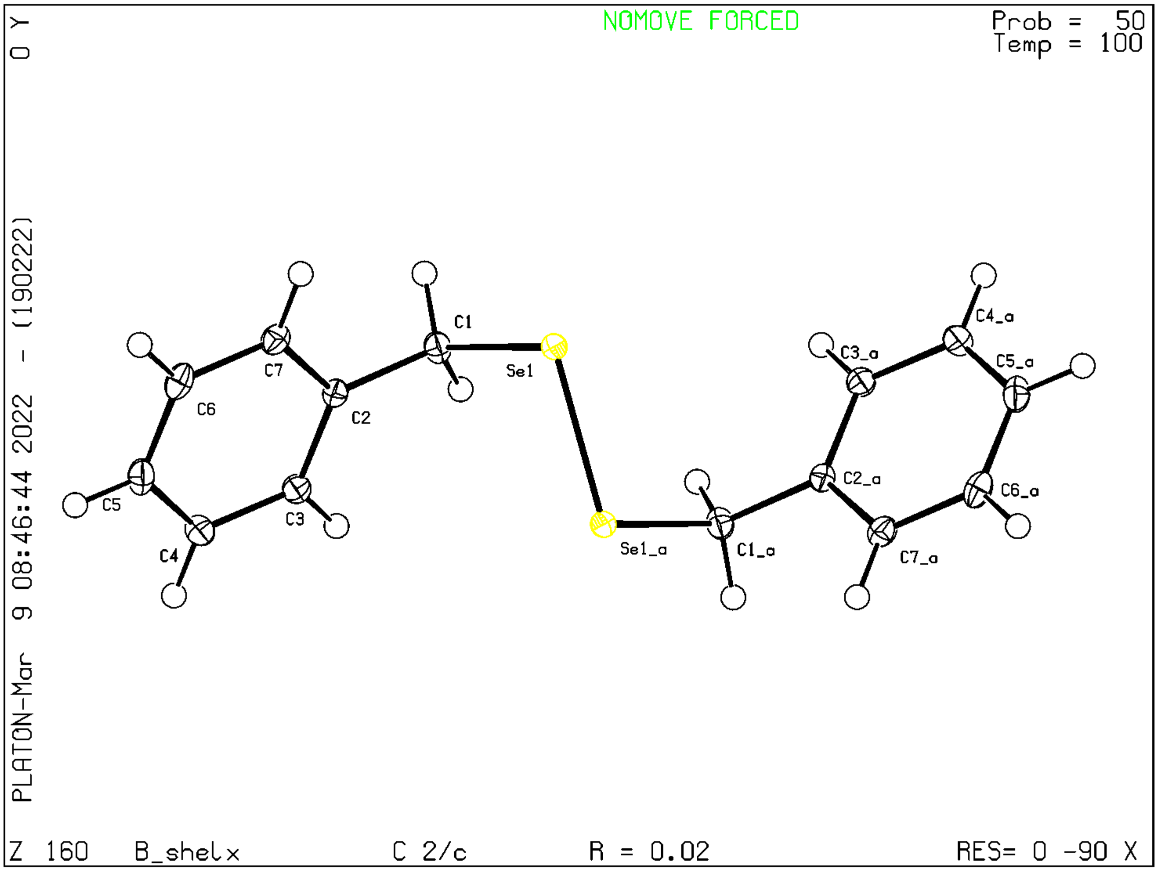

checkCIF/PLATON report

Structure factors have been supplied for datablock(s) C_shelx

THIS REPORT IS FOR GUIDANCE ONLY. IF USED AS PART OF A REVIEW PROCEDURE FOR PUBLICATION, IT SHOULD NOT REPLACE THE EXPERTISE OF AN EXPERIENCED CRYSTALLOGRAPHIC REFEREE.

No syntax errors found. CIF dictionary Interpreting this report

Datablock: C_shelx

Bond precision: C-C = 0.0008 A Wavelength=0.56086

Cell:	a=13.7123(18)	b=8.0051(11)	c=11.4849(15)
	alpha=90	beta=99.260(5)	gamma=90
Temperature:	100 K		

	Calculated	Reported
Volume	1244.3(3)	1244.3(3)
Space group	C 2/c	C 2/c
Hall group	-C 2yc	-C 2yc
Moiety formula	C14 H14 Se2	C14 H14 Se2
Sum formula	C14 H14 Se2	C14 H14 Se2
Mr	340.17	340.17
Dx,g cm-3	1.816	1.816
Z	4	4
Mu (mm-1)	3.135	3.135
F000	664.0	664.0
F000’	665.94	
h,k,lmax	30,17,25	30,17,25
Nref	7206	7123
Tmin,Tmax	0.383,0.572	0.327,0.440
Tmin’	0.355	

Correction method= # Reported T Limits: Tmin=0.327 Tmax=0.440
AbsCorr = MULTI-SCAN

Data completeness= 0.988 Theta(max)= 38.663

R(reflections)= 0.0205(5741) wR2(reflections)= 0.0672(7123)

S = 1.110 Npar= 73

```
The following ALERTS were generated. Each ALERT has the format
       test-name_ALERT_alert-type_alert-level.
Click on the hyperlinks for more details of the test.
```

Alert level G

```
PLAT883_ALERT_1_G No Info/Value for _atom_sites_solution_primary .     Please Do !
PLAT912_ALERT_4_G Missing # of FCF Reflections Above STh/L=  0.600         83 Note
PLAT978_ALERT_2_G Number C-C Bonds with Positive Residual Density.          6 Info
```

```
   0 ALERT level A = Most likely a serious problem - resolve or explain
   0 ALERT level B = A potentially serious problem, consider carefully
   0 ALERT level C = Check. Ensure it is not caused by an omission or oversight
   3 ALERT level G = General information/check it is not something unexpected

   1 ALERT type 1 CIF construction/syntax error, inconsistent or missing data
   1 ALERT type 2 Indicator that the structure model may be wrong or deficient
   0 ALERT type 3 Indicator that the structure quality may be low
   1 ALERT type 4 Improvement, methodology, query or suggestion
   0 ALERT type 5 Informative message, check
```

It is advisable to attempt to resolve as many as possible of the alerts in all categories. Often the minor alerts point to easily fixed oversights, errors and omissions in your CIF or refinement strategy, so attention to these fine details can be worthwhile. In order to resolve some of the more serious problems it may be necessary to carry out additional measurements or structure refinements. However, the purpose of your study may justify the reported deviations and the more serious of these should normally be commented upon in the discussion or experimental section of a paper or in the "special_details" fields of the CIF. checkCIF was carefully designed to identify outliers and unusual parameters, but every test has its limitations and alerts that are not important in a particular case may appear. Conversely, the absence of alerts does not guarantee there are no aspects of the results needing attention. It is up to the individual to critically assess their own results and, if necessary, seek expert advice.

Publication of your CIF in IUCr journals

A basic structural check has been run on your CIF. These basic checks will be run on all CIFs submitted for publication in IUCr journals (*Acta Crystallographica*, *Journal of Applied Crystallography*, *Journal of Synchrotron Radiation*); however, if you intend to submit to *Acta Crystallographica Section C* or *E* or *IUCrData*, you should make sure that full publication checks are run on the final version of your CIF prior to submission.

Publication of your CIF in other journals

Please refer to the *Notes for Authors* of the relevant journal for any special instructions relating to CIF submission.

PLATON version of 19/02/2022; check.def file version of 19/02/2022

Datablock C_shelx - ellipsoid plot

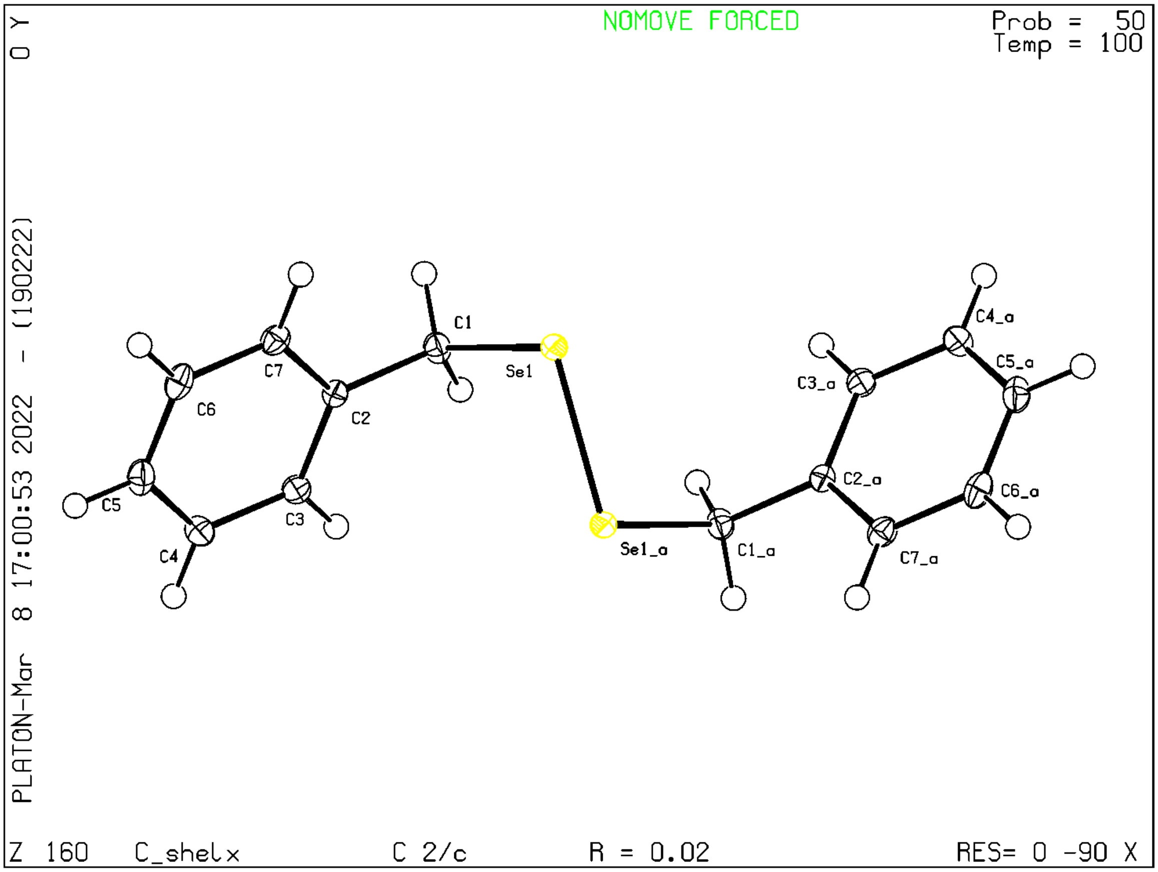

checkCIF/PLATON report

Structure factors have been supplied for datablock(s) d_shelx

THIS REPORT IS FOR GUIDANCE ONLY. IF USED AS PART OF A REVIEW PROCEDURE FOR PUBLICATION, IT SHOULD NOT REPLACE THE EXPERTISE OF AN EXPERIENCED CRYSTALLOGRAPHIC REFEREE.

No syntax errors found. CIF dictionary Interpreting this report

Datablock: d_shelx

```
Bond precision:   C-C = 0.0008 A          Wavelength=0.56086

Cell:            a=13.7199(11)     b=8.0119(6)        c=11.4766(9)
                 alpha=90          beta=99.301(2)     gamma=90
Temperature:     100 K

                Calculated                   Reported
Volume          1244.95(17)                  1244.95(17)
Space group     C 2/c                        C 2/c
Hall group      -C 2yc                       -C 2yc
Moiety formula  C14 H14 Se2                  C14 H14 Se2
Sum formula     C14 H14 Se2                  C14 H14 Se2
Mr              340.17                       340.17
Dx,g cm-3       1.815                        1.815
Z               4                            4
Mu (mm-1)       3.134                        3.134
F000            664.0                        664.0
F000'           665.94
h,k,lmax        30,17,25                     30,17,25
Nref            7204                         7197
Tmin,Tmax       0.324,0.534                  0.376,0.440
Tmin'           0.285

Correction method= # Reported T Limits: Tmin=0.376 Tmax=0.440
AbsCorr = MULTI-SCAN

Data completeness= 0.999           Theta(max)= 38.644

                                                      wR2(reflections)=
R(reflections)= 0.0209( 5996)                         0.0735( 7197)

S = 1.098                   Npar= 73
```

```
The following ALERTS were generated. Each ALERT has the format
       test-name_ALERT_alert-type_alert-level.
Click on the hyperlinks for more details of the test.
```

Alert level B

```
PLAT094_ALERT_2_B Ratio of Maximum / Minimum Residual Density ....       4.32 Report
```

Author Response: This results from the radiation damage.

Alert level C

```
PLAT911_ALERT_3_C Missing FCF Refl Between Thmin & STh/L=    0.600           5 Report
PLAT939_ALERT_3_C Large Value of Not (SHELXL) Weight Optimized S .       10.36 Check
PLAT971_ALERT_2_C Check Calcd Resid. Dens.  0.59Ang From Se1             1.66 eA-3
```

Alert level G

```
PLAT019_ALERT_1_G _diffrn_measured_fraction_theta_full/*_max < 1.0      0.997 Report
PLAT883_ALERT_1_G No Info/Value for _atom_sites_solution_primary .     Please Do !
PLAT912_ALERT_4_G Missing # of FCF Reflections Above STh/L=  0.600          2 Note
PLAT913_ALERT_3_G Missing # of Very Strong Reflections in FCF ....          1 Note
PLAT933_ALERT_2_G Number of HKL-OMIT Records in Embedded .res File          4 Note
PLAT978_ALERT_2_G Number C-C Bonds with Positive Residual Density.          7 Info
```

```
   0 ALERT level A = Most likely a serious problem - resolve or explain
   1 ALERT level B = A potentially serious problem, consider carefully
   3 ALERT level C = Check. Ensure it is not caused by an omission or oversight
   6 ALERT level G = General information/check it is not something unexpected

   2 ALERT type 1 CIF construction/syntax error, inconsistent or missing data
   4 ALERT type 2 Indicator that the structure model may be wrong or deficient
   3 ALERT type 3 Indicator that the structure quality may be low
   1 ALERT type 4 Improvement, methodology, query or suggestion
   0 ALERT type 5 Informative message, check
```

It is advisable to attempt to resolve as many as possible of the alerts in all categories. Often the minor alerts point to easily fixed oversights, errors and omissions in your CIF or refinement strategy, so attention to these fine details can be worthwhile. In order to resolve some of the more serious problems it may be necessary to carry out additional measurements or structure refinements. However, the purpose of your study may justify the reported deviations and the more serious of these should normally be commented upon in the discussion or experimental section of a paper or in the "special_details" fields of the CIF. checkCIF was carefully designed to identify outliers and unusual parameters, but every test has its limitations and alerts that are not important in a particular case may appear. Conversely, the absence of alerts does not guarantee there are no aspects of the results needing attention. It is up to the individual to critically assess their own results and, if necessary, seek expert advice.

Publication of your CIF in IUCr journals

A basic structural check has been run on your CIF. These basic checks will be run on all CIFs submitted for publication in IUCr journals (*Acta Crystallographica*, *Journal of Applied Crystallography*, *Journal of Synchrotron Radiation*); however, if you intend to submit to *Acta Crystallographica Section C* or *E* or *IUCrData*, you should make sure that full publication checks are run on the final version of your CIF prior to submission.

Publication of your CIF in other journals

Please refer to the *Notes for Authors* of the relevant journal for any special instructions relating to CIF submission.

PLATON version of 19/02/2022; check.def file version of 19/02/2022

Datablock d_shelx - ellipsoid plot

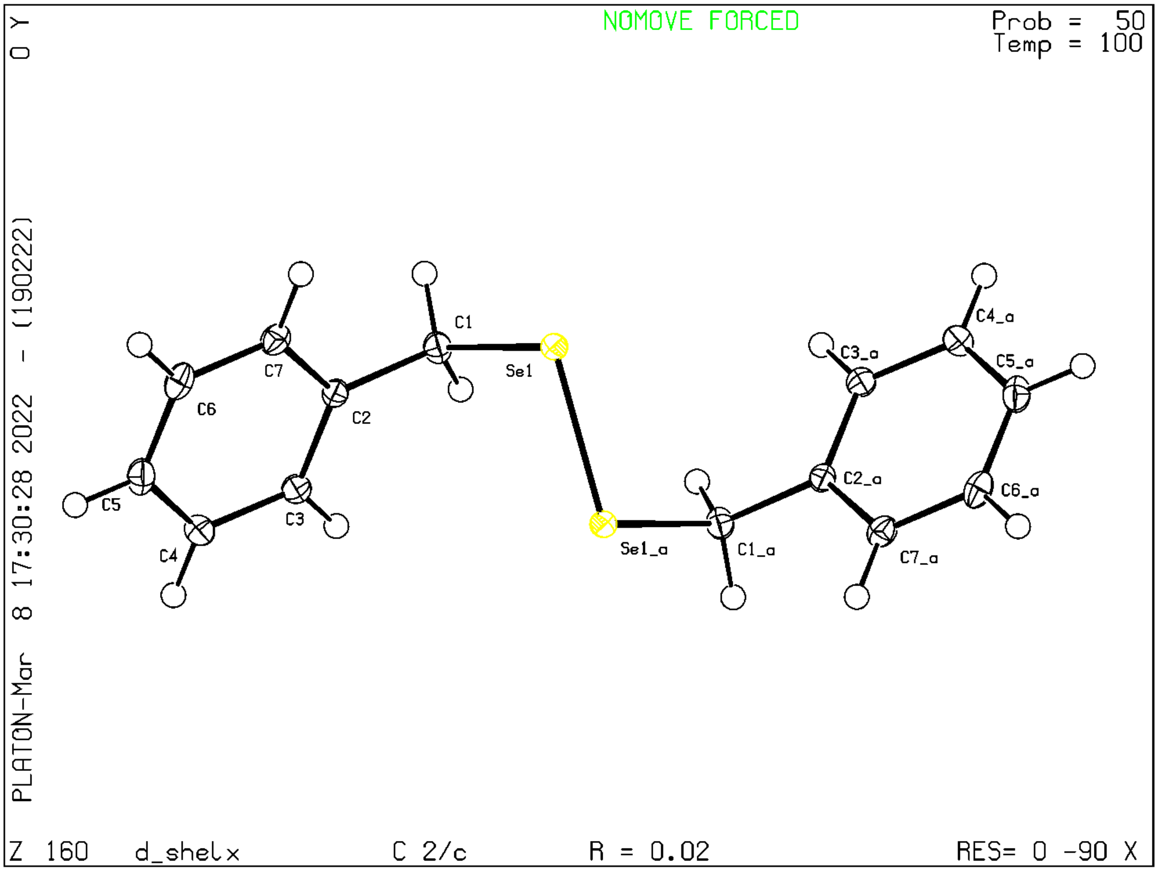

checkCIF/PLATON report

Structure factors have been supplied for datablock(s) E_shelx

THIS REPORT IS FOR GUIDANCE ONLY. IF USED AS PART OF A REVIEW PROCEDURE FOR PUBLICATION, IT SHOULD NOT REPLACE THE EXPERTISE OF AN EXPERIENCED CRYSTALLOGRAPHIC REFEREE.

No syntax errors found. CIF dictionary Interpreting this report

Datablock: E_shelx

Bond precision:	C-C = 0.0006 A		Wavelength=0.56086
Cell:	a=13.7247(7)	b=8.0053(4)	c=11.4831(6)
	alpha=90	beta=99.2776(16)	gamma=90
Temperature:	100 K		

	Calculated	Reported
Volume	1245.15(11)	1245.15(11)
Space group	C 2/c	C 2/c
Hall group	-C 2yc	-C 2yc
Moiety formula	C14 H14 Se2	C14 H14 Se2
Sum formula	C14 H14 Se2	C14 H14 Se2
Mr	340.17	340.17
Dx,g cm-3	1.815	1.815
Z	4	4
Mu (mm-1)	3.133	3.133
F000	664.0	664.0
F000'	665.94	
h,k,lmax	30,17,25	30,17,25
Nref	7181	7171
Tmin,Tmax	0.324,0.534	0.329,0.440
Tmin'	0.286	

Correction method= # Reported T Limits: Tmin=0.329 Tmax=0.440
AbsCorr = MULTI-SCAN

Data completeness= 0.999 Theta(max)= 38.578

R(reflections)= 0.0168(5765) wR2(reflections)= 0.0544(7171)

S = 1.102 Npar= 73

The following ALERTS were generated. Each ALERT has the format
test-name_ALERT_alert-type_alert-level.
Click on the hyperlinks for more details of the test.

Alert level B

PLAT230_ALERT_2_B Hirshfeld Test Diff for Se1 --C1 . 8.1 s.u.

Author Response: This results from the radiation damage.

Alert level C

PLAT911_ALERT_3_C Missing FCF Refl Between Thmin & STh/L= 0.600 2 Report

Alert level G

PLAT883_ALERT_1_G No Info/Value for _atom_sites_solution_primary . Please Do !
PLAT912_ALERT_4_G Missing # of FCF Reflections Above STh/L= 0.600 8 Note
PLAT978_ALERT_2_G Number C-C Bonds with Positive Residual Density. 7 Info

0 **ALERT level A** = Most likely a serious problem - resolve or explain
1 **ALERT level B** = A potentially serious problem, consider carefully
1 **ALERT level C** = Check. Ensure it is not caused by an omission or oversight
3 **ALERT level G** = General information/check it is not something unexpected

1 ALERT type 1 CIF construction/syntax error, inconsistent or missing data
2 ALERT type 2 Indicator that the structure model may be wrong or deficient
1 ALERT type 3 Indicator that the structure quality may be low
1 ALERT type 4 Improvement, methodology, query or suggestion
0 ALERT type 5 Informative message, check

It is advisable to attempt to resolve as many as possible of the alerts in all categories. Often the minor alerts point to easily fixed oversights, errors and omissions in your CIF or refinement strategy, so attention to these fine details can be worthwhile. In order to resolve some of the more serious problems it may be necessary to carry out additional measurements or structure refinements. However, the purpose of your study may justify the reported deviations and the more serious of these should normally be commented upon in the discussion or experimental section of a paper or in the "special_details" fields of the CIF. checkCIF was carefully designed to identify outliers and unusual parameters, but every test has its limitations and alerts that are not important in a particular case may appear. Conversely, the absence of alerts does not guarantee there are no aspects of the results needing attention. It is up to the individual to critically assess their own results and, if necessary, seek expert advice.

Publication of your CIF in IUCr journals

A basic structural check has been run on your CIF. These basic checks will be run on all CIFs submitted for publication in IUCr journals (*Acta Crystallographica*, *Journal of Applied Crystallography*, *Journal of Synchrotron Radiation*); however, if you intend to submit to *Acta Crystallographica Section C* or *E* or *IUCrData*, you should make sure that full publication checks are run on the final version of your CIF prior to submission.

Publication of your CIF in other journals

Please refer to the *Notes for Authors* of the relevant journal for any special instructions relating to CIF submission.

PLATON version of 19/02/2022; check.def file version of 19/02/2022

Datablock E_shelx - ellipsoid plot

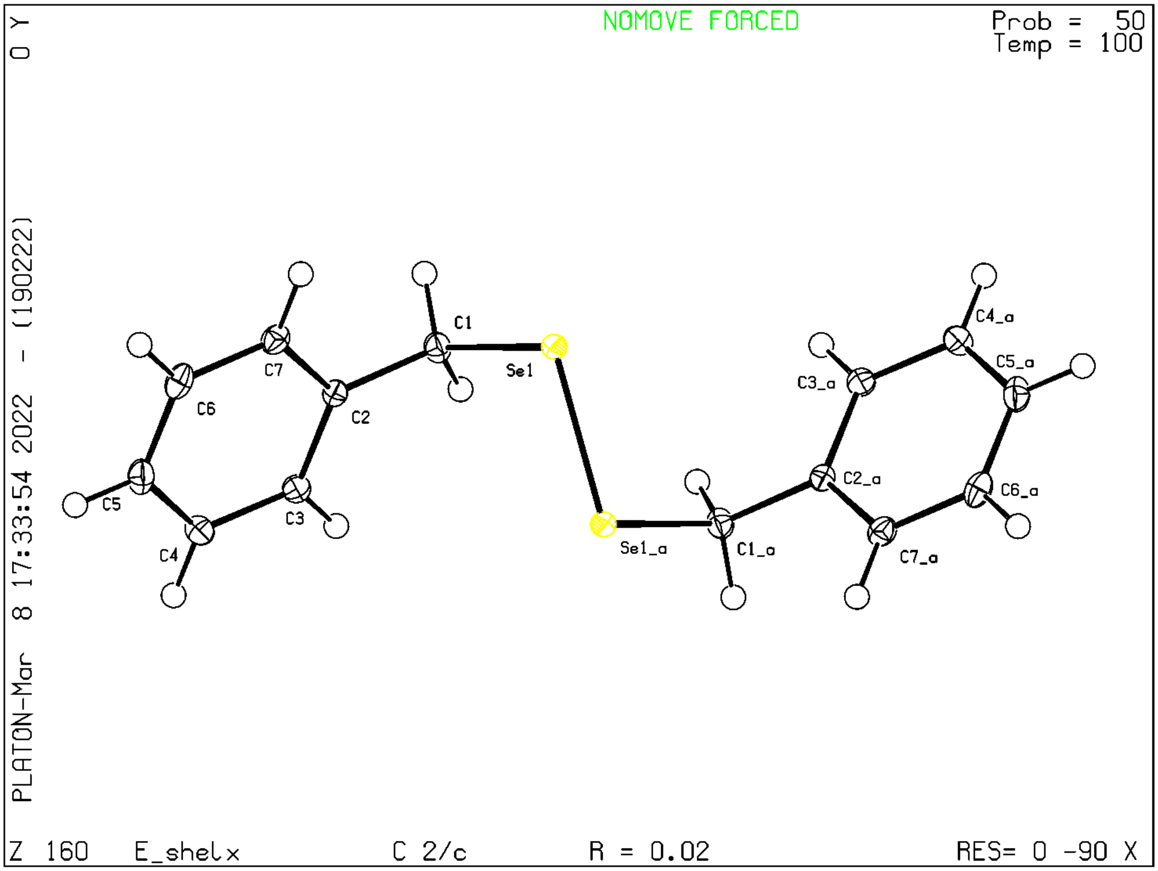

checkCIF/PLATON report

Structure factors have been supplied for datablock(s) F_shelx

THIS REPORT IS FOR GUIDANCE ONLY. IF USED AS PART OF A REVIEW PROCEDURE FOR PUBLICATION, IT SHOULD NOT REPLACE THE EXPERTISE OF AN EXPERIENCED CRYSTALLOGRAPHIC REFEREE.

No syntax errors found. CIF dictionary Interpreting this report

Datablock: F_shelx

Bond precision:	C-C = 0.0008 A		Wavelength=0.51360
Cell:	a=13.7299(6)	b=8.0135(4)	c=11.4875(5)
	alpha=90	beta=99.2957(15)	gamma=90
Temperature:	100 K		

	Calculated	Reported
Volume	1247.31(10)	1247.31(10)
Space group	C 2/c	C 2/c
Hall group	-C 2yc	-C 2yc
Moiety formula	C14 H14 Se2	C14 H14 Se2
Sum formula	C14 H14 Se2	C14 H14 Se2
Mr	340.17	340.17
Dx,g cm-3	1.811	1.811
Z	4	4
Mu (mm-1)	2.463	2.463
F000	664.0	664.0
F000'	666.26	
h,k,lmax	30,17,25	30,17,25
Nref	7182	7171
Tmin,Tmax	0.412,0.611	0.387,0.437
Tmin'	0.373	

Correction method= # Reported T Limits: Tmin=0.387 Tmax=0.437
AbsCorr = MULTI-SCAN

Data completeness= 0.998 Theta(max)= 34.803

R(reflections)= 0.0193(5330) wR2(reflections)= 0.0677(7171)

S = 1.115 Npar= 73

The following ALERTS were generated. Each ALERT has the format
test-name_ALERT_alert-type_alert-level.
Click on the hyperlinks for more details of the test.

Alert level A

```
RADNT01_ALERT_1_A  The radiation type should contain one of the following
            *  'Cu K\a'
            *  'Mo K\a'
            *  'Ag K\a'
            *  'Ga K\a'
            *  'Co K\a'
            *  neutron
            *  synchrotron
```

Author Response: The data were collected with an Excillum Metaljet with Indium radiation.

Alert level C

```
PLAT911_ALERT_3_C Missing FCF Refl Between Thmin & STh/L=    0.600          6 Report
```

Alert level G

```
ABSMU01_ALERT_1_G  Calculation of _exptl_absorpt_correction_mu
                not performed for this radiation type.
PLAT019_ALERT_1_G _diffrn_measured_fraction_theta_full/*_max < 1.0      0.994 Report
PLAT883_ALERT_1_G No Info/Value for _atom_sites_solution_primary .     Please Do !
PLAT910_ALERT_3_G Missing # of FCF Reflection(s) Below Theta(Min).          3 Note
PLAT912_ALERT_4_G Missing # of FCF Reflections Above STh/L=  0.600          2 Note
PLAT933_ALERT_2_G Number of HKL-OMIT Records in Embedded .res File          6 Note
PLAT978_ALERT_2_G Number C-C Bonds with Positive Residual Density.          7 Info
PLAT984_ALERT_1_G The Se-f'=    0.2829 Deviates from the B&C-Value     0.2841 Check
PLAT985_ALERT_1_G The Se-f"=    1.2803 Deviates from the B&C-Value     1.2780 Check
```

1 **ALERT level A** = Most likely a serious problem - resolve or explain
0 **ALERT level B** = A potentially serious problem, consider carefully
1 **ALERT level C** = Check. Ensure it is not caused by an omission or oversight
9 **ALERT level G** = General information/check it is not something unexpected

6 ALERT type 1 CIF construction/syntax error, inconsistent or missing data
2 ALERT type 2 Indicator that the structure model may be wrong or deficient
2 ALERT type 3 Indicator that the structure quality may be low
1 ALERT type 4 Improvement, methodology, query or suggestion
0 ALERT type 5 Informative message, check

It is advisable to attempt to resolve as many as possible of the alerts in all categories. Often the minor alerts point to easily fixed oversights, errors and omissions in your CIF or refinement strategy, so attention to these fine details can be worthwhile. In order to resolve some of the more serious problems it may be necessary to carry out additional measurements or structure refinements. However, the purpose of your study may justify the reported deviations and the more serious of these should normally be commented upon in the discussion or experimental section of a paper or in the "special_details" fields of the CIF. checkCIF was carefully designed to identify outliers and unusual parameters, but every test has its limitations and alerts that are not important in a particular case may appear. Conversely, the absence of alerts does not guarantee there are no aspects of the results needing attention. It is up to the individual to critically assess their own results and, if necessary, seek expert advice.

Publication of your CIF in IUCr journals

A basic structural check has been run on your CIF. These basic checks will be run on all CIFs submitted for publication in IUCr journals (*Acta Crystallographica*, *Journal of Applied Crystallography*, *Journal of Synchrotron Radiation*); however, if you intend to submit to *Acta Crystallographica Section C* or *E* or *IUCrData*, you should make sure that full publication checks are run on the final version of your CIF prior to submission.

Publication of your CIF in other journals

Please refer to the *Notes for Authors* of the relevant journal for any special instructions relating to CIF submission.

PLATON version of 19/02/2022; check.def file version of 19/02/2022

Datablock F_shelx - ellipsoid plot

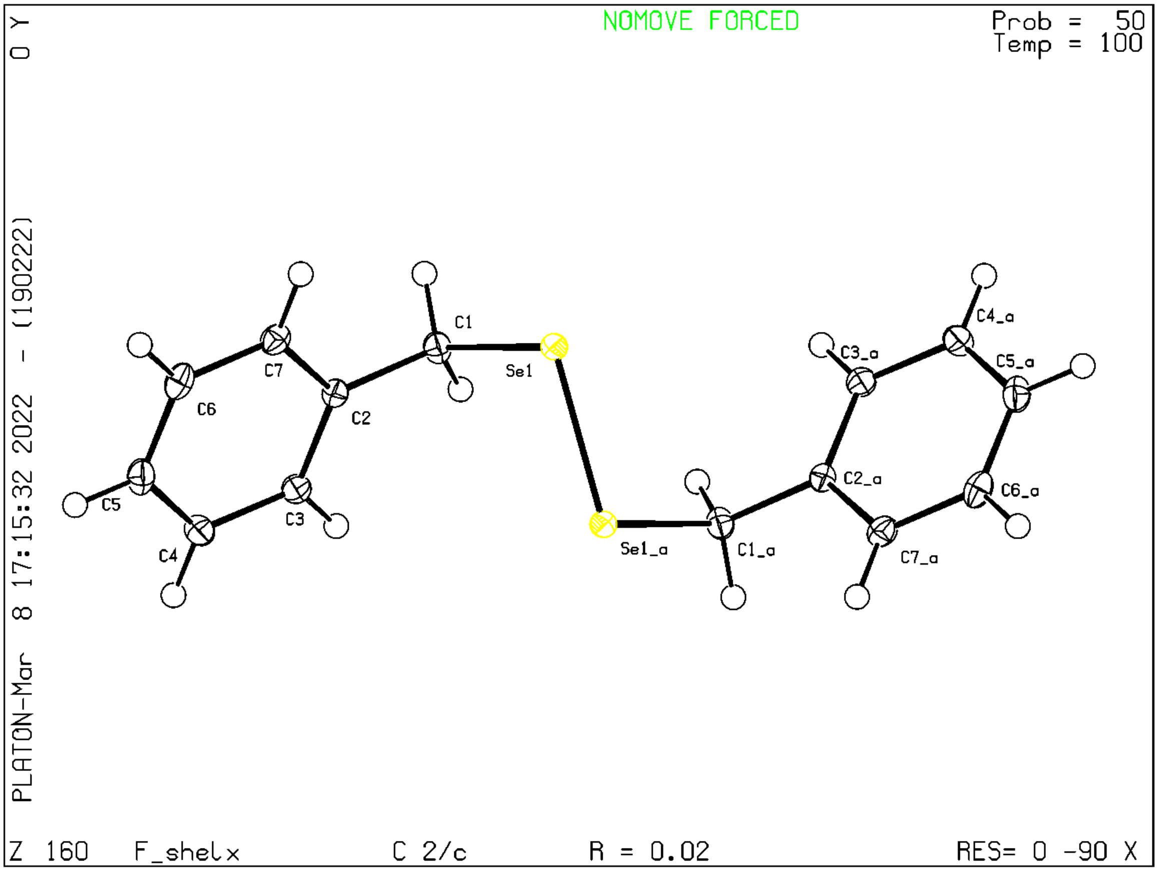

Supplement: Supplementary file 3 — Supporting Information [file ANIE-61-0-s001.pdf]
